# Decoding Drug Resistance in Pancreatic Cancer: A Subcellular Structure Perspective

**DOI:** 10.3390/biology15070574

**Published:** 2026-04-02

**Authors:** Xiaowen Li, Hao Lyu, Yixin Wu, Anyi Chen, Guifang Wu, Rui Zhang, Shuai Xiao, Dong Guo, Qi Zhang, Chaojun Yan, Jingfeng Tang, Cefan Zhou

**Affiliations:** 1 National “111” Center for Cellular Regulation and Molecular Pharmaceutics, Hubei University of Technology, Wuhan 430068, China; xiaowenli0202@163.com (X.L.);; 2 Cooperative Innovation Center of Industrial Fermentation (Ministry of Education & Hubei Province), Hubei Key Laboratory of Industrial Microbiology, Hubei University of Technology, Wuhan 430068, China

**Keywords:** pancreatic ductal adenocarcinoma, subcellular structure, drug resistance, cell homeostasis, tumorigenesis

## Abstract

Pancreatic ductal adenocarcinoma (PDAC) remains one of the most lethal malignancies, with drug resistance representing the primary barrier to effective treatment. Current research has largely focused on individual signaling pathways or isolated organelle functions, yet a comprehensive understanding of how these subcellular structures coordinate to drive resistance remains lacking. This review synthesizes current knowledge through the perspective of subcellular structural homeostasis, the dynamic balance maintained by intracellular organelles. We examine how key subcellular structures, the cell membrane, mitochondria, endoplasmic reticulum, ribosomes, lysosomes, exosomes, and stress granules, undergo functional remodeling to promote drug resistance. It is crucial that these organelles do not work independently but form an integrated and dynamic communication network. Mitochondria serve as the intracellular signaling hub, integrating calcium signals, metabolic progress, and stress responses, while exosomes function as intercellular messengers that spread the anti-drug-resistant phenotype between cells. This framework reveals why targeting individual structures often fails and highlights the therapeutic potential of disrupting inter-organelle communication. We discuss emerging clinical strategies targeting subcellular structures and identify critical knowledge gaps, including the need for non-invasive biomarkers and combination approaches that target multiple network nodes. By shifting the focus from isolated organelles to their coordinated interplay, this review offers a new paradigm for overcoming drug resistance in PDAC.

## Simple Summary

Pancreatic cancer is a highly lethal malignancy and rapidly acquires chemotherapy resistance through adaptive cellular responses that help cells withstand treatment stress. Within each cell, organelles such as mitochondria (powerhouses), lysosomes (recycling hubs), and the endoplasmic reticulum (protein synthesis factory) normally work together to maintain homeostasis. During treatment, these organelles are dynamically rewired to support cancer cell survival. Importantly, they not only function in isolation, but they also engage in constant bidirectional crosstalk, forming a collaborative network that drastically enhances survival. In this review, we delineate this regulatory network, highlighting mitochondria as the central hub and exosomes as intercellular messengers. Understanding this communication system opens new therapeutic possibilities. Disrupting critical communication nodes between organelles could resensitize cancer cells to therapy and improve clinical outcomes for pancreatic ductal adenocarcinoma patients.

## 1. Introduction

Pancreatic ductal adenocarcinoma (PDAC), the predominant histologic subtype of pancreatic cancer, accounts for greater than 90% of all pancreatic malignancies [1,2]. As a highly aggressive digestive malignancy, PDAC is characterized by an extremely poor prognosis, with a 5-year overall survival rate of approximately 13% for patients in the United States [3,4]. Currently, PDAC ranks as the 3rd leading cause of cancer-related mortality in the US, and projections indicate it will become the second leading cause of cancer-related death by 2030 [5]. This severe and growing disease burden stems from three interrelated core factors: the lack of effective early diagnostic modalities, the intricate pathogenesis of PDAC, and widespread therapeutic resistance to existing treatments [4,6].

The difficult-to-access anatomical location of the pancreas, the non-specific clinical symptoms in the early stage of PDAC, and the lack of early diagnostic biomarkers with both high specificity and sensitivity result in the majority of patients being diagnosed only when the disease has progressed to locally advanced stages or distant metastasis. Only 10% to 15% of patients have the opportunity for curative surgical resection at initial diagnosis. The most commonly used serum biomarker in clinical practice, CA19-9, has poor diagnostic sensitivity for early PDAC. Insufficient early diagnostic capability is a key bottleneck limiting the improvement of prognosis for PDAC patients [5,7]. This lack of early diagnostic opportunities means curative surgical resection is not an option for the vast majority of patients, leaving systemic therapy as the mainstay of clinical management, yet the efficacy of these treatments is severely limited by the universal and refractory nature of PDAC therapeutic resistance.

Current standard-of-care treatments for PDAC include surgical resection, chemotherapy, radiotherapy, and immunotherapy. Although complete surgical resection is a primary treatment options, PDAC is mostly diagnosed at an advanced or metastatic stage, rendering only 15–20% of patients eligible for curative-intent surgery [5]. Even among patients who undergo successful resection, the majority will develop disease recurrence or distant metastasis postoperatively [8,9]. Unlike multiple solid tumors that achieve durable clinical benefits from immune checkpoint inhibitors, PDAC harbors a broadly immunosuppressive phenotype, leading to very limited efficacy of immunotherapeutic approaches in most patients. Accordingly, systemic chemotherapy remains the backbone of clinical management for both resectable and advanced PDAC. Gemcitabine was the first chemotherapeutic agent approved for advanced PDAC, yet its efficacy as monotherapy is modest, with a median overall survival of merely 6.7 months [10]. For metastatic PDAC, combination regimens including gemcitabine plus nanoparticle albumin-bound paclitaxel (nab-paclitaxel) and modified FOLFIRINOX have shown superior survival benefits versus gemcitabine monotherapy in phase III clinical trials [11]. However, the broad clinical application of these intensive regimens is largely limited by severe treatment-related toxicities and high economic costs [12]. Most critically, the rapid and universal development of resistance to existing treatments, including chemotherapy, targeted therapy and immunotherapy, remains the major barrier to improving long-term outcomes in PDAC [8,13].

The profound drug resistance in PDAC is driven by adaptive mechanisms operating at multiple biological levels, which have been extensively investigated in preclinical and clinical studies. Notably, the root of drug resistance in PDAC is inextricably linked to its intricate pathogenesis: the multistep evolution from pre-neoplastic lesions (such as pancreatic intraepithelial neoplasia) to invasive carcinoma is driven by the sequential accumulation of genomic alterations, most prominently oncogenic KRAS activation (present in >90% of cases) and inactivating mutations in tumor suppressor genes tumor protein p53 (TP53), cyclin dependent kinase inhibitor 2A (CDKN2A), and mothers against decapentaplegic homolog 4 (SMAD4) [1,2,5]. These oncogenic drivers not only initiate and sustain tumor progression, but also fundamentally reshape the biological properties of PDAC cells to mediate drug resistance. The mutant KRAS reprograms mitochondrial metabolism through upregulation of glutamine utilization and de novo lipid synthesis, while loss of SMAD4 enhances mitophagy flux to clear damaged mitochondria and enable evasion of apoptotic cell death [14,15]. Driven by these oncogenic genomic alterations, PDAC cells undergo extensive metabolic reprogramming, including the Warburg effect, glutamine addiction, and dysregulated lipid metabolism. These biochemical adaptations to drive uncontrolled proliferation and survival of PDAC under therapeutic stress by maintaining energy homeostasis and biosynthetic capacity in the nutrient-deprived and hypoxic tumor microenvironment (TME) [3,10,16]. These genotype-driven metabolic and organellar adaptations are further coordinated through interconnected signaling pathway networks. Hyperactivation of the MAPK/ERK pathway, a hallmark of KRAS-mutant PDAC, directly modulates mitochondrial dynamics to promote pathological fission [17]. The PI3K/serine-threonine kinase (AKT) pathway integrates upstream signals from the cell membrane, endoplasmic reticulum (ER), and mitochondria to suppress apoptotic signaling and promote cell survival [18,19]. Meanwhile, the unfolded protein response (UPR), serves as a master regulator of ER homeostasis and communicates stress signals to mitochondria and stress granules (SGs) [20,21]. Beyond these cell-intrinsic adaptive mechanisms, the dense desmoplastic stroma pathognomonic of PDAC further amplifies drug resistance through inherent biophysical effects [22].

In cancer cells, subcellular structures serve as central mediators of cellular metabolism, stress responses, and signal transduction, frequently displaying aberrant, dysregulated, and hyperactive activities. Subcellular structures activate adaptive responses to maintain homeostasis under conditions such as environmental and therapeutic stress. For instance, mitochondrial quality control (MQC), UPR, ER-phagy, and the dynamic assembly of SGs have been reported as key mechanisms driving drug resistance in PDAC [14,23,24,25]. Therefore, the principal objectives of the drug treatments are not only to directly kill cancer cells, but also to overcome these adaptive survival mechanisms and destroy these supportive signaling networks, so that resistant cells are re-sensitive to existing therapies. Although significant progress has been made in genetic, metabolic, and signaling pathways underlying PDAC drug resistance, a crucial research gap remains. Current studies predominantly focus on individual signaling pathways or isolated organelle functions, and the role of interactions among subcellular structures in PDAC drug resistance still requires clarification.

This review outlines the mechanisms underlying dysregulation of subcellular structural homeostasis and its role in driving PDAC drug resistance. It summarizes the crosstalk among subcellular structures, with a focus on mitochondria as intracellular signaling hubs and exosomes as key intercellular communication messengers, highlighting their crucial roles in the PDAC drug resistance network. We also discuss clinically advanced therapeutic strategies targeting subcellular structures and promising future research directions. This article deepens our understanding of the pivotal role of subcellular structural homeostasis in PDAC drug resistance and presents prospective insights into innovative therapeutic strategies aimed at overcoming resistance in PDAC.

## 2. Cell Membrane

**Figure 1:**
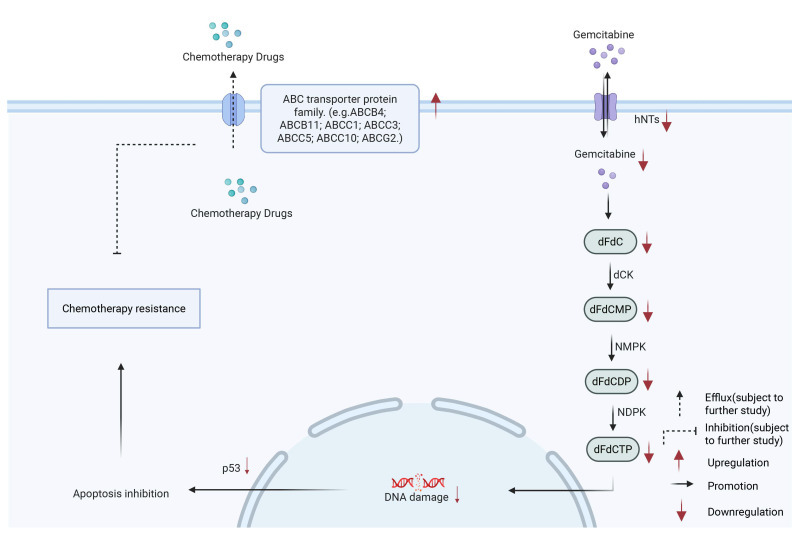
Role of the cell membrane in drug resistance of PDAC. Dysregulation of membrane transporter proteins contributes to chemoresistance in PDAC by coordinately altering drug influx and efflux across the cell membrane. Decreased expression of drug uptake transporters (e.g., hENT1) reduces the intracellular accumulation of chemotherapeutic agents, thereby limiting drug availability at intracellular targets [27]. Meanwhile, upregulation or functional activation of efflux transporters from the ABC family, including ABCB1 and ABCG2, actively pumps drugs out of the cell [28,29,30,31]. The combined effects of reduced drug uptake and enhanced drug efflux disrupt intracellular drug homeostasis, ultimately decreasing drug efficacy and promoting chemoresistance in PDAC. Created with BioRender by Li, X. (2026). https://BioRender.com/6nvlha5 (accessed on 30 March 2026).

The distinctive pathological features of the pancreatic cancer microenvironment present substantial barriers to the effective penetration of most therapeutic agents into PDAC cells. Despite these challenges, chemotherapy remains a primary treatment strategy for pancreatic cancer. Chemotherapeutic agents exert their therapeutic effects by entering pancreatic cancer cells via transporters located on the cell membrane [22]. As the core barrier of transmembrane transport, aberrant expression or hyperactivity of membrane proteins such as ion channels, transporters, and exchangers underlies impaired drug transport and drug resistance in PDAC, thereby further compounding therapeutic challenges. Dysregulation of protein expression involved in drug uptake and efflux at the cell membrane constitutes a significant mechanism underlying PDAC drug resistance (Figure 1).

Gemcitabine enters cells through nucleoside transporters located on the cell membrane, including human concentrative nucleoside transporters (hCNTs) and human equilibrative nucleoside transporters (hENTs), which contribute to its concentration and transmembrane transport. Once transported into the cell, gemcitabine is phosphorylated by kinases such as deoxycytidine kinase (dCK), nucleoside monophosphate kinase (NMPK), and nucleoside diphosphate kinase (NDPK), and converted into its active metabolites, namely gemcitabine diphosphate (dFdCDP) and triphosphate (dFdCTP) [26]. Limiting the intracellular uptake of gemcitabine by reducing the expression of human equilibrative nucleoside transporter 1 (hENT1) on the cell membrane is a known mechanism of drug resistance. Studies have shown that the survival rates of patients with low expression of nucleoside transporter proteins is significantly reduced [27].

The ATP-binding cassette (ABC) transport protein family on the cell membrane is the core functional protein that mediates the external discharge of drugs. Such proteins can actively pump intracellular drugs to the extracellular or vesicles through ATP energy supply, thus substantially reducing the effective concentration of intracellular drugs. Studies have demonstrated that ATP-binding cassette subfamily B member 4 (ABCB4), ATP-binding cassette subfamily B member 11 (ABCB11), ATP-binding cassette subfamily C member 1 (ABCC1), ATP-binding cassette subfamily C member 3 (ABCC3), ATP-binding cassette subfamily C member 5 (ABCC5), ATP-binding cassette subfamily C member 10 (ABCC10), ATP-binding cassette subfamily G member 2 (ABCG2) and other ABC family members show significant upregulation in PDAC [28]. ATP-binding cassette subfamily B member 1 (ABCB1) (also known as multidrug resistance protein 1 (MDR1) or P-glycoprotein (P-gp)) is physiologically expressed in various tissues, including the pancreas, correlates with poor clinical outcomes, and plays a critical role in mediating resistance to paclitaxel (including nab-paclitaxel) [29]. Evidence indicates that paclitaxel serves as a specific substrate for ABCB1, whereby the overexpression of ABCB1 on the cell membrane facilitates ATP-dependent transmembrane efflux of intracellular drug molecules, ultimately contributing to chemoresistance in PDAC cells. However, the role of ABCB1 in gemcitabine resistance remains controversial. Although some studies suggest that ABCB1 may be implicated in resistance to gemcitabine, supported by molecular docking analyses indicating gemcitabine as a potential substrate of ABCB1 [30], ABCB1 has not been found to be overexpressed in gemcitabine-resistant models, and pharmacological inhibition by verapamil failed to enhance gemcitabine sensitivity [31].

Collectively, the cell membrane serves as a central adaptive hub for PDAC drug resistance. Its role primarily focuses on coordinating membrane protein regulation processes, including membrane dynamics, drug uptake and efflux, changes in membrane protein expression, and metabolic remodeling. Through these mechanisms, the cell membrane maintains the balance of material exchange between the cell and its environment, allowing the cell to evade drug-induced death. This capability positions the cell membrane as a crucial regulator of cancer cell survival, highlighting the metabolic flexibility of membrane proteins as a promising therapeutic target for overcoming PDAC chemoresistance.

## 3. Mitochondria

**Figure 2:**
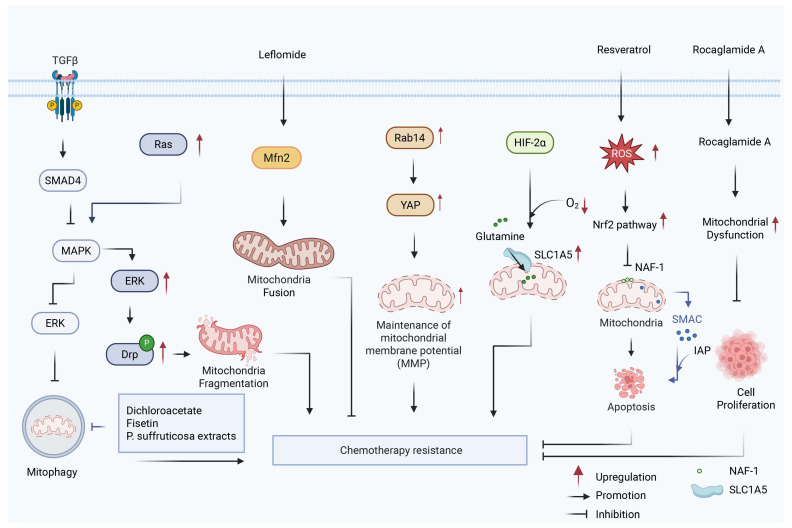
Role of mitochondrial homeostasis in drug resistance of PDAC. Dysregulation of mitochondrial regulatory processes contributes to chemoresistance in PDAC by coordinately modulating mitochondrial dynamics, mitophagy, metabolic reprogramming, and apoptosis. The Oncogenic Ras/MAPK signaling promotes Drp1-mediated excessive mitochondrial fission [17], whereas SMAD4 deletion enhances mitophagy via MAPK/ERK activation [14], collectively maintaining mitochondrial quality control under stress conditions. Metabolic reprogramming involving NRF2 activation [40,41] and glutamine metabolism mediated by SLC1A5 variants [42] further supports mitochondrial bioenergetic and redox homeostasis. Meanwhile, altered apoptotic regulation, including upregulation of BCL2 [18] and modulation of mitochondrial proteins such as SMAC [36] and NAF-1 [40], suppresses apoptosis and preserves mitochondrial function. The coordinated interplay of enhanced mitochondrial fission, increased mitophagy, metabolic reprogramming, and suppressed apoptosis collectively sustains tumor cell survival and promotes chemoresistance in PDAC. Created with BioRender by Li, X. (2026). https://BioRender.com/1xskn3u (accessed on 30 March 2026).

Mitochondria, as the “energy factories” of cells, provide cells with directly utilizable energy molecules ATP, which are the direct energy source of cellular life activities. Calcium (Ca^2+^) signaling plays a crucial role in the physiological regulation of pancreatic cell function. In normal pancreatic cells, Ca^2+^ signals are triggered by hormones or neurotransmitters, leading to the release of Ca^2+^ from the ER. This Ca^2+^ signaling is closely linked to mitochondrial energy metabolism, where mitochondria rapidly take up Ca^2+^ and enhance ATP production through oxidative phosphorylation (OXPHOS). ATP generated by mitochondria then supports Ca^2+^ transporters, maintaining intracellular Ca^2+^ homeostasis. However, there are still significant gaps in the research on the relationship between Ca^2+^ signaling and ATP production, particularly in pancreatic cancer cells. While some studies have explored the link between Ca^2+^ signaling and tumor growth, the mechanisms by which Ca^2+^ disrupts ATP metabolism and contributes to chemoresistance remain poorly understood and require further investigation [32,33] MQC is a regulatory system based on the dynamic characteristics of mitochondria and their ability to maintain mitochondrial quality and function under multiple cellular stresses. MQC maintains mitochondrial homeostasis by coordinating various processes such as mitochondrial biogenesis, fission, fusion, protein degradation, and mitophagy [16,34]. MQC is also a key factor contributing to the development of chemoresistance in PDAC (Figure 2).

Mitochondrial dynamics, particularly the balance between fission and fusion, plays a crucial role in cancer cell proliferation [35]. Activation of the oncogenic Ras or mitogen-activated protein kinase (MAPK) pathway triggers excessive mitochondrial fission and fragmentation through extracellular signal-regulated kinase 2 (ERK2)-mediated phosphorylation of dynamin-related protein 1 (Drp1), thereby promoting tumor growth. Inhibiting Drp1-dependent mitochondrial fission effectively suppresses MAPK-driven malignancies, including PDAC, highlighting targeted mitochondrial fission inhibition as a promising therapeutic strategy [17]. Furthermore, transforming growth factor beta (TGFβ) signaling and SMAD4 loss also contribute to PDAC chemoresistance. Specifically, SMAD4 deletion enhances mitophagy by activating MAPK/ERK signaling to clear damaged mitochondria, while TGFβ signaling promotes cell survival by inducing epithelial–mesenchymal transition (EMT) alongside mitochondrial fragmentation [14].

In addition to mitochondrial dynamics and mitophagy, apoptotic regulation centered on mitochondria represents another critical determinant of PDAC chemosensitivity. Rab14, a member of the small GTPase RAS family, upregulates B-cell lymphoma 2 (BCL2) expression by increasing Yes-associated protein (YAP) levels, and BCL2 can prevent mitochondrial membrane potential decrease induced by gemcitabine [18]. Conversely, activation of the mitochondrial protein second mitochondria-derived activator of caspases (SMAC) triggers its translocation to the cytoplasm, where it binds to inhibitor of apoptosis proteins (IAPs) to promote apoptosis [36]. Consistent with this mechanism, the SMAC analog SW IV-134 significantly enhances PDAC sensitivity to gemcitabine and inhibits tumor growth in preclinical models by activating the apoptotic pathway [37,38].

Mitochondrial metabolic reprogramming and redox homeostasis further exacerbate PDAC chemoresistance. Dysregulated Ca^2+^ signaling results in sustained cytosolic Ca^2+^ levels and altered inter-organelle Ca^2+^ transfer in PDAC. These disruptions in Ca^2+^ homeostasis drive metabolic adaptations that facilitate tumor growth and survival. Moreover, the activation of pro-survival pathways by dysregulated Ca^2+^ signaling positions impaired Ca^2+^ signaling as a critical factor in PDAC drug resistance, offering potential targets for therapeutic intervention. Notably, oncogenic KRAS mutation disrupts mitochondrial Ca^2+^ handling by altering MAM integrity, specifically impairing the GRP75-IP_3_R-VDAC1 complex. This reduces mitochondrial Ca^2+^ uptake and compromises OXPHOS [39]. This forces PDAC cells to rely on glycolysis for ATP production, but the resulting ATP deficiency further impairs the activity of Ca^2+^ clearance pumps (PMCA and SERCA), creating a vicious cycle of cytosolic Ca^2+^ overload and metabolic dysfunction [32]. Nutrient deprivation autophagy factor-1 (NAF-1) is an outer mitochondrial membrane protein involved in regulating calcium metabolism, anti-apoptotic activity, and anti-autophagic activity. Resveratrol has been shown to inhibit PDAC cell proliferation by inducing reactive oxygen species (ROS) accumulation and activating the nuclear factor erythroid 2-related factor 2 (NRF2) signaling pathway, which in turn promotes apoptosis and enhances sensitivity to gemcitabine [40]. In KRAS-mutant PDAC, the transcription factor NRF2 drives metabolic reprogramming and enhances glutamine metabolism, thereby promoting chemoresistance, a process that is correlated with a poor prognosis [41]. Moreover, a variant of the mitochondrial glutamine transporter solute carrier family 1 member 5 (SLC1A5) is induced by hypoxia-inducible factor 2 alpha (HIF-2α) under hypoxic conditions. This variant facilitates glutamine transport into the mitochondria for metabolism, thereby supporting energy production and antioxidant defense, which ultimately confers gemcitabine resistance upon PDAC cells [42].

In light of this, mitochondria-targeted therapeutic strategies are emerging as a novel approach to overcoming drug resistance in PDAC. Research has demonstrated that inhibiting Drp1 or activating mitofusin 2 (Mfn2) to reverse mitochondrial fragmentation and enhance mitochondrial fusion can effectively suppress OXPHOS and induce mitophagy, thereby significantly inhibiting tumor progression. Leflunomide, an FDA-approved drug for arthritis, has been shown to upregulate Mfn2 expression, promote mitochondrial fusion, and demonstrate therapeutic efficacy in spontaneous cancer mouse models of PDAC [43]. Furthermore, transglutaminase 2 (TGM2) mediates gemcitabine resistance in PDAC by reprogramming glutamine metabolism, thereby activating mitophagy. This metabolic adaptation is sustained through a positive feedback loop involving the purinergic receptor P2X7 (P2RX7), ultimately promoting chemoresistance [44]. Additionally, the bioactive molecule Rocaglamide A has been found to induce mitochondrial dysfunction in PDAC cells, suppress tumor growth, and promote apoptosis, indicating its potential as a therapeutic agent [45].

The regulatory role of mitochondria in PDAC chemoresistance is primarily centered on the coordination of MQC processes, including mitochondrial dynamics, mitophagy, apoptosis regulation, and metabolic reprogramming. Through these mechanisms, PDAC cells maintain mitochondrial integrity and promote cell survival under therapeutic pressure. A unifying theme emerging from these interconnected processes is mitochondrial metabolic plasticity: the ability of mitochondria to dynamically reprogram substrate utilization, redox balance, and energy production in response to stress. As discussed above, oncogenic KRAS mutations disrupt mitochondrial Ca^2+^ handling, forcing PDAC cells to rely on glycolysis and creating a vicious cycle of Ca^2+^ overload and metabolic dysfunction. Concurrently, NRF2-driven metabolic reprogramming and hypoxia-induced adaptations enhance glutamine metabolism and antioxidant defenses, enabling PDAC cells to withstand chemotherapy-induced oxidative stress. By integrating these quality control mechanisms, mitochondrial metabolic plasticity emerges as a central determinant of PDAC cell fate under therapeutic stress. Targeting this plasticity represents a promising therapeutic strategy to overcome chemoresistance in PDAC.

## 4. Endoplasmic Reticulum

**Figure 3:**
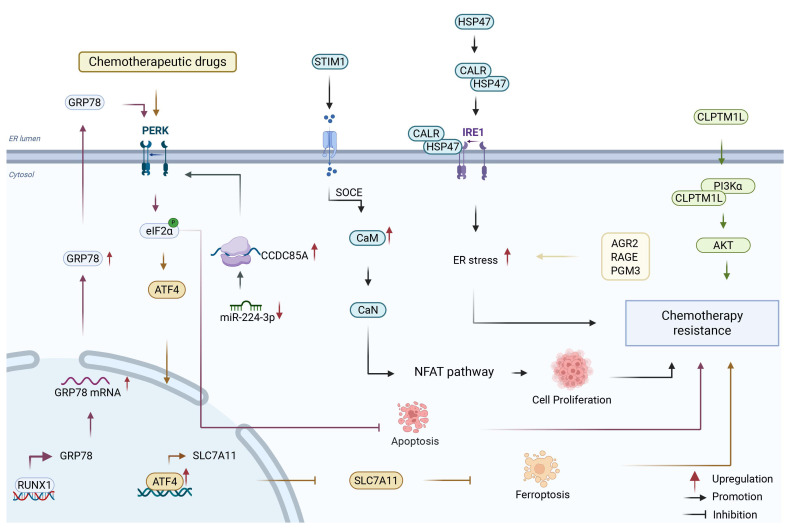
Role of ER-mediated regulation in drug resistance of PDAC. Dysregulation of ER stress signaling contributes to chemoresistance in PDAC by coordinately activating adaptive stress response pathways and pro-survival signaling. Activation of the GRP78/PERK/eIF2α axis is activated by RUNX1 to inhibit chemotherapy-induced apoptosis [21], while PERK/ATF4 signaling upregulates SLC7A1 suppress ferroptosis [54]. In parallel, CLPTM1L interacts with GRP78 and PI3Kα to activate the PI3K/AKT survival signaling pathway [19] and HSP47 interacts with CALR and IRE1α to induce ER stress [51]. Additionally, CAFs modulate the CCDC85A-GRP78/94-PERK-ATF4 axis via miR-224-3p, promoting ER stress adaptation [52]. Co-amplification of PGM3 [55] and RRM1/STIM1 further enhances Ca^2+^ signaling, facilitating adaptation to ER stress and activation of the NFAT pathway [56]. The adaptation to ER stress, inhibition of apoptosis and ferroptosis, and activation of pro-survival signaling pathways maintain cellular homeostasis under therapeutic stress, promoting the development of chemotherapy resistance in PDAC. Created with BioRender by li, X. (2026). https://BioRender.com/6he6bg7 (accessed on 30 March 2026).

The ER serves as a critical site for protein synthesis, folding, and post-translational modification. It also functions as a major intracellular Ca^2+^ store that maintains Ca^2+^ homeostasis, which is an essential process for pancreatic physiological function [20,32]. In normal pancreatic acinar cells, Ca^2+^ homeostasis is tightly regulated: hormonal (e.g., cholecystokinin, CCK) or neurotransmitter (e.g., acetylcholine, ACh) stimulation activates the Gαq/phospholipase Cβ pathway to generate inositol 1,4,5-trisphosphate (IP_3_), which binds to IP_3_ receptors (IP_3_R) on the ER membrane to trigger Ca^2+^ release. ER Ca^2+^ depletion subsequently activates store-operated Ca^2+^ entry (SOCE) via stromal interaction molecule 1 (STIM1)-Orai1 complexes to refill the ER Ca^2+^ pool, while plasma membrane Ca^2+^-ATPases (PMCA) and ER SERCA pumps clear excess cytosolic Ca^2+^ to avoid cytotoxicity [32,33,46]. Disturbances such as the accumulation of misfolded proteins, dysregulated Ca^2+^ homeostasis, hypoglycemia, and hypoxia can trigger ER stress [47,48], a condition that subsequently activates the unfolded protein response (UPR). The UPR is predominantly regulated by three ER transmembrane sensors: inositol-requiring enzyme 1 (IRE1), protein kinase R (PKR)-like ER kinase (PERK), and activating transcription factor 6 (ATF6), the detailed molecular mechanisms of which have been extensively reviewed [49]. Persistent ER stress has been implicated in the pathogenesis of various diseases, notably cancer [50]. Notably, the disruption of ER homeostasis is intricately linked to the emergence of drug resistance in PDAC (Figure 3). Based on the accumulated evidence, we will introduce the drug resistance mechanism mediated by ER from the three perspectives of adapting to cell stress, inhibiting cell apoptosis and reprogramming of metabolism.

Accumulating evidence suggests that ER stress plays a critical role in promoting the adaptive survival of PDAC cells. The transcription factor RUNX family transcription factor 1 (RUNX1) facilitates the activation of the PERK/eIF2α signaling pathway by upregulating the ER stress sensor protein glucose-regulated protein 78kDa (GRP78). This upregulation enhances the capacity of cancer cells to adapt to stress, consequently inhibiting apoptosis induced by chemotherapeutic agents [21]. The ER stress-related protein cleft lip and palate transmembrane protein 1-like (CLPTM1L) interacts with GRP78 and phosphoinositide 3-kinase alpha (PI3Kα), culminating in the activation of the PI3K/AKT survival signaling pathway, which in turn confers drug resistance to tumor cells [19]. Additionally, the molecular chaperone heat shock protein 47 (HSP47) interacts with the ER calcium regulatory protein calreticulin (CALR) and Inositol-requiring enzyme 1 alpha (IRE1α) to induce ER stress, thereby promoting chemoresistance in PDAC [51]. CAFs modulate the function of the CCDC85A-GRP78/94-PERK-ATF4 axis by diminishing the supply of the protective microRNA miR-224-3p, thereby enhancing cancer cell resistance to ER stress [52]. Furthermore, the ER-associated protein anterior gradient 2 (AGR2), a dithiol isomerase highly expressed in PDAC, is extensively involved in tumor metastasis, chemoresistance, and adaptive ER stress responses. Specifically, AGR2 has been reported to regulate tunicamycin-induced ER stress and influence cell viability following gemcitabine treatment in PDAC [53].

The administration of chemotherapeutic agents is associated with the activation of the PERK pathway, wherein the nuclear translocation of activating transcription factor 4 (ATF4) leads to the increased expression of the ferroptosis inhibitory protein solute carrier family 7 member 11 (SLC7A11). This signaling event suppresses ferroptosis and thereby contributes to the development of chemoresistance [53]. Another investigation indicates that the core ER stress regulatory protein GRP78, together with the transcription factor specificity protein 1 (SP1), can inhibit the efflux activity of ABC transporters, thus mediating chemoresistance in PDAC [54]. Together, these studies illustrate how ER stress signaling supports PDAC survival by suppressing cell death pathways.

Drug resistance is intricately associated with alterations in cellular metabolism. Research has demonstrated that the key enzyme phosphoglucomutase 3 (PGM3) within the hexosamine biosynthetic pathway is markedly upregulated in drug-resistant cells. Inhibition of PGM3 leads to a reduction in protein glycosylation, activation of the UPR, and attenuation of the epidermal growth factor receptor (EGFR)/AKT signaling pathway, thereby overcoming resistance to gemcitabine [55]. Furthermore, Ca^2+^ homeostasis serves as a critical link between ER stress and drug resistance. Investigations have revealed co-amplification of ribonucleotide reductase catalytic subunit M1 (RRM1) and STIM1 in PDAC cells resistant to gemcitabine. STIM1, as an ER calcium sensor protein, is primarily activated when ER Ca^2+^ stores are depleted, typically triggered by hormonal or neurotransmitter stimulation. This depletion prompts STIM1 to translocate to the cell membrane, where it interacts with Orai1 to facilitate SOCE. The STIM1-mediated elevation of cytoplasmic Ca^2+^ signaling mitigates ER stress and activates the nuclear factor of activated T-cells (NFAT) pathway, thereby promoting cell survival and contributing to chemoresistance [56]. These findings highlight that ER-associated metabolic rewiring and calcium signaling represent additional layers of adaptation that support tumor survival by maintaining bioenergetic balance and stress tolerance.

Several intervention strategies aimed at modulating ER homeostasis have demonstrated considerable efficacy in mitigating resistance in PDAC. The natural compound ursolic acid has been shown to induce ER stress by suppressing the expression of the receptor for advanced glycosylation end-products (RAGE), consequently modulating apoptotic and autophagic pathways and reversing RAGE-mediated chemoresistance [57]. Additionally, the GDMCN2 (Gemcitabine-Derived Multifunctional Carbon Nitride Nanocage 2) nanocage system developed by Zhao et al. facilitates the release of ROS, triggers Ca^2+^ efflux, and induces ER stress during targeted therapy, thereby enhancing the sensitivity of PDAC cells to gemcitabine [58]. It is important to note that under certain conditions, the augmentation of ER stress may enhance drug sensitivity; for instance, PDAC cells harboring a melanoma inhibitory activity 2 (MIA2) mutation exhibit elevated levels of ER to nucleus signaling 1 (ERN1) and show increased susceptibility to gemcitabine [59]. Overall, these therapeutic observations suggest that carefully modulating ER stress, either enhancing or relieving it depending on context, may help overcome PDAC drug resistance.

In summary, ER stress plays a crucial role in chemoresistance in PDAC through three interconnected mechanisms: promoting cellular adaptation to stress, enhancing resistance to apoptosis, and driving metabolic reprogramming. A deeper understanding of the complex role of ER stress in PDAC will help develop more effective therapeutic strategies to overcome chemoresistance and provides a foundation for developing drugs targeting ER homeostasis.

## 5. Ribosome

**Figure 4:**
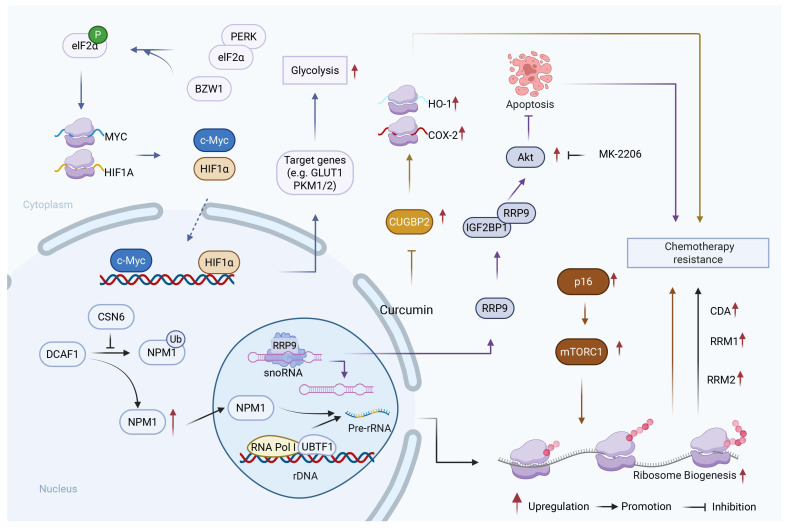
Role of ribosome-mediated translational regulation in drug resistance of PDAC. Dysregulation of ribosome biogenesis and translational control contributes to chemoresistance in PDAC by coordinately enhancing selective translation of resistance-associated proteins, activating pro-survival signaling pathways, and promoting drug efflux. CSN6-driven stabilization of NPM1 enhances ribosome biogenesis, thereby increasing the translational efficiency of resistance-associated genes (e.g., CDA and RRM1/2) [62]. RRP9 binding to IGF2BP1 activates AKT survival signaling [63], while BZW1 promotes the synthesis of HIF1α and c-Myc via eIF2α phosphorylation and IRES-dependent translation [61], supporting metabolic adaptation under stress. Sustained mTORC1 activity upon p16 loss further maintains ribosomal synthesis and translational capacity [64,65], whereas ERK overactivation induces nucleolar stress and adaptive responses [66]. The combined effects of enhanced ribosome biogenesis, selective translational reprogramming, activation of pro-survival signaling pathways, and exosome-mediated drug efflux collectively sustain tumor cell survival and ultimately promote chemoresistance in PDAC. Created with BioRender by Li, X. (2026). https://BioRender.com/2awob43 (accessed on 30 March 2026).

Ribosome homeostasis is critical for cellular function and survival under both normal and stress conditions. This homeostasis is achieved through the coordinated actions of ribosome-associated quality control (RQC), ribophagy, and ribosome biogenesis. Ribosome homeostasis is maintained through coordinated quality control, degradation, and biogenesis (Figure 4). RQC resolves stalled or collided ribosomes during translation elongation by recruiting ZNF598, NEMF, and LTN1 for proteasomal degradation of aberrant polypeptides. Complementing RQC, ribophagy selectively delivers entire ribosomes to lysosomes via receptors such as NUFIP1 and Rpl12, recycling nucleotides and amino acids under nutrient deprivation. Ribosome biogenesis assembles functional ribosomal units and is dynamically regulated by stress-sensing pathways including SAPK, AMPK, and p53. Together, these interconnected mechanisms ensure protein homeostasis and metabolic adaptation under cellular stress.

Although the key role of RQC and ribophagy in regulating the homeostatic state of Ribosome has been discovered, their research in the course of tumors and drug resistance is still limited. The chemotherapeutic agent 5-fluorouracil (5-FU) and its metabolites induce ribosomal stalling in pancreatic cancer cells. Notably, ZNF598 knockout cells exhibit significantly enhanced sensitivity to 5-FU, indicating that RQC-mediated resolution of stalled ribosomes represents an adaptive mechanism that enables cancer cells to withstand drug-induced translational stress and contributes to chemoresistance [60]. Furthermore, in response to cellular stress, ribosomes selectively reprogram protein synthesis through RQC-related pathways. For instance, acting as an adaptive effector of PERK, basic leucine zipper and W2 domains 1 (BZW1) facilitates the reprogramming of glycolysis and enhances tumor cell survival under metabolic stress by promoting the phosphorylation of eIF2α and enabling internal ribosomal entry site (IRES)-dependent translation of hypoxia-inducible factor 1 alpha (HIF-1α) or cellular myelocytomatosis oncogene (c-Myc) [61].

The upregulation of ribosome biogenesis represents a key adaptive mechanism employed by tumors to counteract chemotherapeutic stress. Notably, COP9 signalosome subunit 6 (COPS6) stabilizes nucleophosmin 1 (NPM1), a critical factor for ribosome biogenesis, by counteracting DCAF1-mediated ubiquitin-dependent degradation. This stabilization promotes ribosome biogenesis and subsequently enhances the translational efficiency of genes implicated in gemcitabine resistance, including those encoding cytidine deaminase (CDA) and ribonucleotide reductase catalytic subunit M1/2 (RRM1/2) [62]. Furthermore, ribosomal RNA processing 9 (RRP9), a protein involved in ribosomal RNA processing, directly interacts with insulin-like growth factor 2 mRNA binding protein 1 (IGF2BP1) to activate the AKT signaling pathway, mitigating DNA damage and suppressing apoptosis, thereby promoting PDAC progression and gemcitabine resistance [63]. The mechanistic target of rapamycin kinase complex 1 (mTORC1) activity is sustained in PDAC, ensuring high translational capacity by promoting both ribosome biogenesis and translational initiation. This helps PDAC cells evade cell cycle arrest induced by cyclin-dependent kinase 4/6 (CDK4/6) inhibitors and continue to synthesize proteins critical for survival, thereby contributing to therapeutic resistance [64,65]. Furthermore, hyperactivation of ERK signaling can provoke nucleolar stress, induce cellular senescence, and thus provide a mechanistic rationale for strategies targeting ribosomal function [66].

Based on the aforementioned mechanisms, various ribosome-targeted therapeutic approaches are currently under investigation. For instance, curcumin has been shown to reverse gemcitabine resistance by upregulating the ribosome-associated RNA-binding protein CUG triplet repeat, RNA binding protein 2 (CUGBP2), thereby downregulating cytoprotective proteins such as heme oxygenase 1 (HO-1) and cyclooxygenase-2 (COX-2) [67].

In conclusion, ribosomes are not only central to protein synthesis but also play a crucial role in regulating PDAC chemoresistance through three interconnected mechanisms: RQC-mediated resolution of translational stress, ribophagy-driven metabolic adaptation, and amplified ribosome biogenesis for enhanced translational capacity. Together, these pathways enable PDAC cells to maintain protein homeostasis, adapt to metabolic stress, and sustain production of pro-survival proteins under therapeutic pressure. A deeper understanding of the complex interactions between ribosome homeostasis and chemotherapy resistance pathways provides valuable insights into potential therapeutic strategies targeting ribosomal function to enhance the efficacy of PDAC treatments.

## 6. Lysosome

Lysosomes are essential cellular structures that facilitate energy production and supply raw materials necessary for cell growth through the degradation of biological macromolecules [68,69,70,71]. The lysosomal lumen contains more than 60 types of acid hydrolases, which primarily function to degrade proteins and diverse substrates derived from multiple sources, playing a critical role in maintaining cellular homeostasis [72]. Lysosomal instability or rupture results in the release of hydrolases, Ca^2+^, and H^+^, which can significantly compromise cellular function [73,74]. Overall, lysosome-mediated mechanisms of therapeutic resistance mainly involve four major processes: metabolic regulation through autophagy-related pathways, lysosome-mediated degradation of drug transporters and regulatory proteins, immune evasion, and lysosomal stability.

Lysosome-mediated selective protein degradation represents a direct mechanism of chemoresistance by reducing drug uptake or stabilizing pro-survival factors. The prolyl isomerase peptidylprolyl cis/trans isomerase, NIMA-interacting 1 (Pin1) facilitates the lysosomal degradation of the gemcitabine transporter ENT1, thereby reducing drug accumulation and promoting chemoresistance. Conversely, the tumor suppressor F-box and WD repeat domain containing 7 (FBW7) enhances gemcitabine efficacy by inhibiting ENT1 degradation, highlighting the regulatory balance governing drug transporter stability [75]. Beyond transporters, the deubiquitinase ubiquitin-specific protease 7 (USP7) inhibits autophagy-lysosome-mediated degradation of insulin-like growth factor 2 mRNA binding protein 2 (IGF2BP2) by removing its ubiquitin modifications. Stabilized IGF2BP2 increases platelet-derived growth factor subunit A (PDGFA) mRNA stability, activating cancer-associated fibroblasts (CAFs) in the TME and ultimately promoting gemcitabine resistance [76]. This mechanism exemplifies how lysosomal activity within cancer cells can indirectly influence stromal cells through paracrine signaling, linking intracellular protein degradation to broader microenvironmental remodeling.

Autophagy is vital for PDAC cell survival and the acquisition of chemoresistance [77], with selective autophagy playing a key role in disease progression [78]. Ferritinophagy, a form of selective autophagy mediated by nuclear receptor coactivator 4 (NCOA4), targets ferritin for lysosomal degradation [79]. Lysosomes serve as conduits for the release of iron from ferritin, which is subsequently transported to mitochondria to meet metabolic demands and counteract therapeutic stress, thereby enhancing cell survival and chemoresistance [80]. This lysosome-mitochondria iron shuttle directly links organellar function to metabolic adaptation, as mitochondrial iron is essential for OXPHOS and Fe-S cluster biogenesis.

Lysosome-dependent metabolic regulation also contributes to resistance against ferroptosis, a form of iron-dependent cell death. The actin-regulatory protein tropomodulin 3 (TMOD3), highly expressed in KRAS-mutant PDAC cells, accelerates autophagosome-lysosome fusion by promoting F-actin polymerization. This process enhances lysosomal degradation of acyl-CoA synthetase long chain family member 4 (ACSL4), a key enzyme that sensitizes cells to ferroptosis by incorporating polyunsaturated fatty acids into membrane phospholipids [78]. Notably, ACSL4 localizes to the ER and mitochondria-associated membranes (MAMs), where its activity influences lipid peroxidation and ferroptosis sensitivity. By degrading ACSL4, lysosomes indirectly modulate MAM function and the ER-mitochondria axis, demonstrating how lysosomal activity can reshape inter-organelle crosstalk to promote drug resistance.

Lysosomal function and integrity play a critical role in modulating immune responses and resistance to immunotherapy. Major histocompatibility complex class I (MHC-I) molecules are recognized by the autophagy receptor next to BRCA1 gene 1 (NBR1), which facilitates their delivery to lysosomes for degradation, thereby promoting immune evasion [81]. Receptor-interacting protein kinase 2 (RIPK2) enhances NBR1 activity, driving lysosomal degradation of MHC-I, impairing tumor antigen presentation and contributing to acquired resistance to immunotherapies, including anti-PD-1 antibody treatment [82]. Conversely, N-myc downstream regulated 1 (NDRG1), a stress response and tumor suppressor gene, counteracts resistance to immune checkpoint blockade by inhibiting lysosomal degradation of MHC-I and upregulating antigen presentation [83]. This pathway connects lysosomal function to the ER (where MHC-I molecules are assembled) and the plasma membrane (where antigen presentation occurs), highlighting the role of lysosomes in regulating immune recognition through organellar crosstalk.

Lysosomal stability is essential for tumor cell survival and determines the mode of cell death in response to therapy. The enzyme cytochrome P450 family 51 subfamily A member 1 (CYP51A1), a key component of the cholesterol biosynthesis pathway, prevents alkaline-induced cell death in PDAC by maintaining lysosomal cholesterol levels. This process activates transmembrane protein 175 (TMEM175)-mediated proton efflux, preserving the acidic lysosomal environment required for optimal hydrolase activity [84].

A bidirectional relationship exists between chemotherapeutic agents and lysosomal function. Gemcitabine increases lysosomal enzyme activity, correlating with dose-dependent increases in acid α-glucosidase (GAA) activity. GAA facilitates lysosomal glucose production, supporting cancer cell survival and contributing to chemoresistance [85]. Additionally, gemcitabine activates transcription factor EB (TFEB), promoting its nuclear translocation and enhancing lysosomal biogenesis and function. Conversely, TFEB knockdown significantly inhibits PDAC cell proliferation [86]. Similarly, mitogen-activated protein kinase kinase (MEK) inhibitors induce TFEB nuclear translocation, leading to increased lysosomal biogenesis and conferring chemoresistance [87].

**Figure 5:**
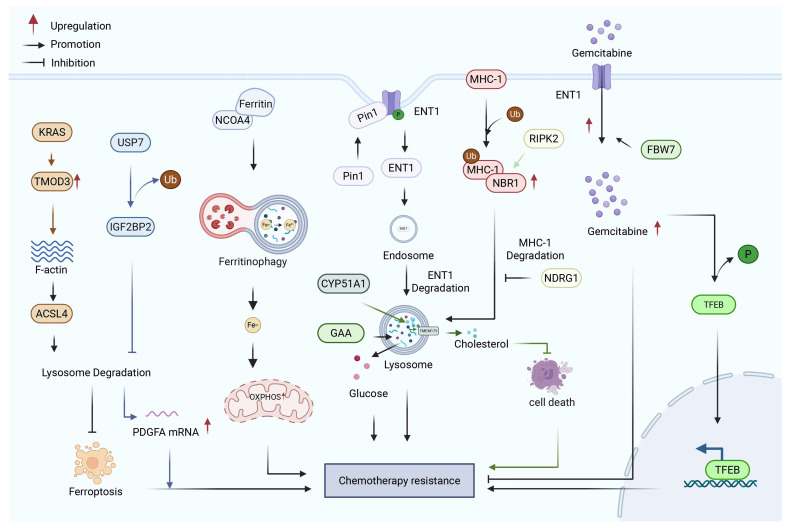
Role of lysosome-mediated regulation in drug resistance of PDAC. Dysregulation of lysosomal function contributes to chemoresistance in PDAC by coordinately modulating metabolic adaptation, selective protein degradation, immune evasion, and lysosomal stability. NCOA4-dependent ferritinophagy promotes iron release to support mitochondrial metabolism [78], while TMOD3-mediated autophagosome-lysosome fusion enhances degradation of ACSL4 to inhibit ferroptosis [80]. Lysosome-dependent protein degradation further contributes to resistance, including Pin1-mediated degradation of ENT1 [75], which impairs drug uptake, and USP7-mediated stabilization of IGF2BP2, leading to activation of CAFs and pro-survival signaling [76]. In parallel, NBR1-mediated lysosomal degradation of MHC-I, facilitates immune evasion [82]. Maintenance of lysosomal stability ensures the acidic lysosomal environment required for cellular adaptation, for example through CYP51A1-mediated regulation of cholesterol homeostasis [84]. In addition, chemotherapeutic agents such as gemcitabine or MEK inhibitors activating TFEB, enhancing lysosomal biogenesis [87]. Lysosome-mediated coordination of metabolic adaptation, selective protein degradation, immune evasion, and lysosomal stability collectively drives chemoresistance in PDAC. Created with BioRender by Li, X. (2026). https://BioRender.com/5yszioc (accessed on 30 March 2026).

Collectively, these findings highlight the critical role of lysosomes as hubs integrating metabolic adaptation, protein degradation, immune modulation, and cell death decisions in PDAC chemoresistance. Through their interactions with mitochondria (iron transfer, mitophagy), the ER (MAMs, ACSL4 localization), and autophagosomes (fusion), lysosomes form an interconnected organellar network that determines cellular responses to therapeutic stress. This comprehensive understanding of lysosomal function emphasizes its potential as a therapeutic target for overcoming PDAC chemoresistance (Figure 5).

## 7. Exosomes

**Figure 6:**
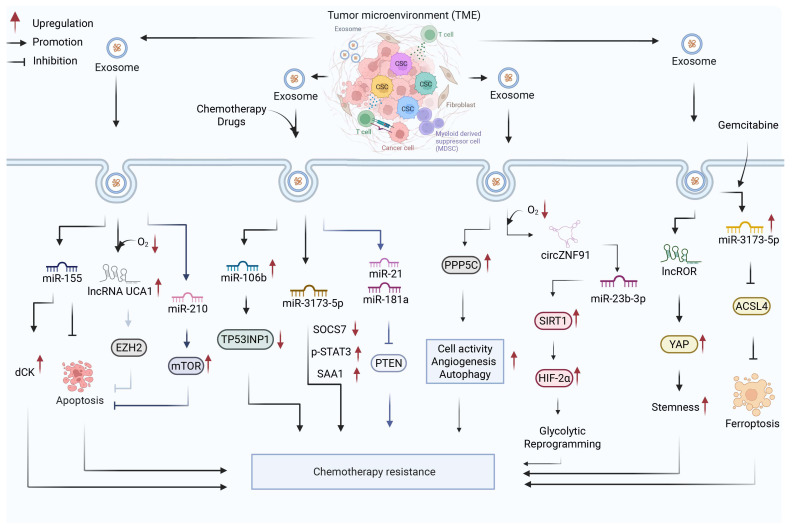
Role of exosome-mediated intercellular communication in drug resistance of PDAC. Exosomes contribute to chemoresistance in PDAC by coordinately mediating intercellular transfer of resistance-associated factors, tumor–stroma communication, and metabolic reprogramming of recipient cells. Exosomes derived from gemcitabine-resistant PDAC cells, CAFs, cancer-associated adipocytes, pancreatic stellate cells, and cancer stem cells deliver diverse bioactive cargos, including proteins, miRNAs, circRNAs, and lncRNAs, to recipient cells [97,98,99,100,101,102,103]. These cargos promote resistance through multiple mechanisms, including inhibition of drug activation (e.g., suppression of dCK) [26,94,95,96,112], activation of pro-survival signaling pathways (e.g., mTOR and AKT) [113], induction of EMT [94,97,112,114,115,116,117], suppression of ferroptosis, and enhancement of stemness. Hypoxia- or stress-induced exosomes also drive metabolic reprogramming in recipient cells, supporting adaptation to therapeutic stress. The coordinated effects of intercellular transfer of resistance-associated factors, microenvironmental remodeling, and metabolic reprogramming mediated by exosomes collectively promote chemoresistance in PDAC. Created with BioRender by Li, X. (2026). https://BioRender.com/qsfmhdr (accessed on 30 March 2026).

Exosomes are a subset of extracellular vesicles (EVs) enriched with bioactive constituents such as proteins, nucleic acids, lipids, and metabolites, playing a critical role within the TME [88]. The resistance of PDAC to gemcitabine predominantly arises from its pronounced genetic heterogeneity and dense stromal microenvironment. In this context, acting as pivotal mediators of intercellular communication, exosomes facilitate the systemic transmission and amplification of chemoresistance by delivering diverse functional molecules [89]. Importantly, once internalized by recipient cells, exosomal cargo can modulate the function of multiple organelles, including mitochondria, ER, lysosomes, and ribosomes, thereby linking donor cell status to recipient cell subcellular homeostasis. Accumulating evidence indicates that transfer of drug-resistant molecules, tumor-matrix communication, and the reprogramming of receptor cell metabolism and organelle function are all important factors for exosome-mediated drug resistance in PDAC (Figure 6).

The transfer of resistance-associated molecules from drug-resistant to drug-sensitive cells via exosomes represents a key mechanism driving the dissemination of chemoresistance in PDAC. Gemcitabine-resistant PDAC cells export proteins and transcripts through exosomes to sensitive counterparts, thereby propagating the resistant phenotype [90,91,92]. For instance, resistant cells secrete exosomes containing ROS detoxification enzymes (SOD2, CAT) and their corresponding mRNAs, while simultaneously delivering miR-155, which downregulates the drug-activating enzyme dCK. This dual mechanism diminishes cytotoxic efficacy and reduces the metabolic activation of gemcitabine [37,93,94,95,96]. Prolonged gemcitabine exposure upregulates miR-155, enhancing anti-apoptotic pathways and promoting exosome secretion, thus establishing a positive feedback loop that accelerates resistance dissemination within the tumor population [37].

Interactions between PDAC cells and stromal components via exosomes constitute another critical axis of chemoresistance. CAFs secrete exosomes enriched with snail family transcriptional repressor 1 (SNAIL) and miRNAs such as miR-21 and miR-181a, which suppress tumor suppressor genes (PTEN, TP53INP1) and promote proliferation and drug resistance in cancer cells [97,98,99]. Similarly, cancer-associated adipocytes and pancreatic stellate cells (PSCs) release exosomes that modulate inflammatory pathways and enhance resistance [100,101,102]. Conversely, PDAC cells release exosomes containing protein phosphatase 5 catalytic subunit (PPP5C) into the microenvironment, stimulating endothelial activity, angiogenesis, and autophagy, collectively contributing to gemcitabine resistance. The regulatory relationship between miR-520-5p and PPP5C influences patient prognosis, underscoring the clinical relevance of this bidirectional communication [103].

Beyond direct molecular transfer and stromal communication, exosomes can remodel the metabolic landscape and organellar function of recipient cells. These effects are mediated through exosomal cargo that targets key regulators of mitochondrial, ER, and lysosomal function. Exosomes also contribute to metabolic and phenotypic reprogramming of recipient cells under stress conditions such as hypoxia. Exosomes secreted by hypoxic PDAC cells transfer circZNF91 and lncROR to cells in normoxic conditions. These molecules enhance resistance to gemcitabine by regulating metabolic pathways such as glycolysis and the Hippo-YAP signaling axis [104,105]. CAF-derived exosomal miR-421 targets SIRT3, a mitochondrial deacetylase, regulating the SIRT3/H3K9Ac/HIF-1α axis and thereby affecting pancreatic cancer cell proliferation and survival [106,107]. SIRT3 downregulation leads to mitochondrial dysfunction and activates HIF-1α-mediated transcriptional reprogramming. Additionally, exosomes derived from PSCs carry miR-4456 and miR-616-3p, which activate AKT signaling by targeting PTEN, indirectly affecting mitochondria-mediated apoptosis regulation [108]. Exosomes secreted by normal fibroblasts carry miR-224-3p, which targets CCDC85A and modulates PERK-mediated ER stress responses. Upon conversion of normal fibroblasts to CAFs, miR-224-3p downregulation relieves CCDC85A suppression, rendering cancer cells resistant to ER stress-induced apoptosis [52]. This mechanism directly links exosomes, the ER stress response, and chemotherapy resistance.

Several strategies have shown promise in counteracting exosome-mediated resistance, including inhibition of exosome biogenesis/secretion (e.g., GW4869), blockade of specific cargo transfer (e.g., miR-365 antagonists), and engineering exosomes as therapeutic delivery vehicles (e.g., Survivin-T34A-loaded exosomes) [98,109,110]. Inhibition of macrophage migration inhibitory factor (MIF) activity also disrupts exosome-mediated immunosuppression and enhances antitumor immunity [111].

Despite these advances, key challenges remain. The precise contribution of exosome-mediated drug efflux to resistance is not fully quantified. Whether pharmacological inhibition of exosome secretion can sustainably enhance chemosensitivity in vivo without triggering compensatory pathways is unclear. Most critically, the molecular mechanisms governing selective cargo packaging remain poorly understood. Addressing these gaps is essential for developing targeted, mechanism-based therapies. In summary, exosomes serve as intercellular hubs that link donor cell status to recipient cell subcellular homeostasis by delivering cargo that directly modulates organelle function of recipient cell. This framework deepens our understanding of exosome biology in drug resistance and highlights the therapeutic potential of targeting exosome biogenesis, secretion, and cargo composition.

## 8. Stress Granules

**Figure 7:**
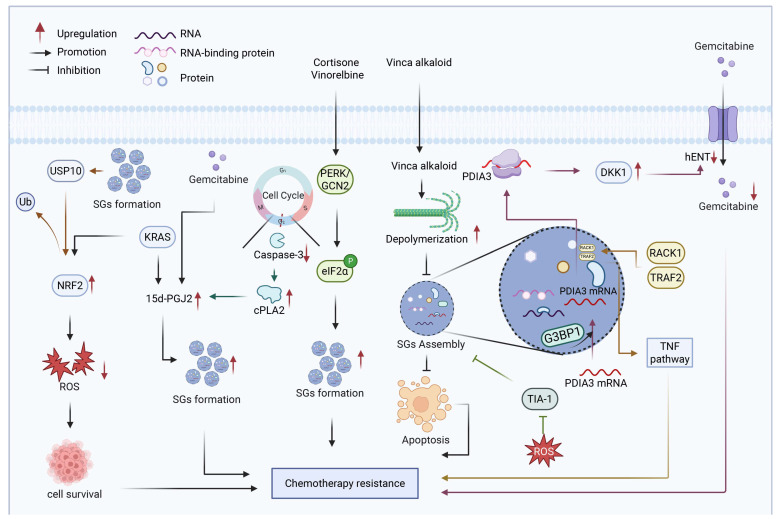
Role of SGs-mediated regulation in drug resistance of PDAC. Dysregulation of SG dynamics contributes to chemoresistance in PDAC by coordinately modulating oxidative stress responses, RNA metabolism, apoptosis signaling, and drug uptake. KRAS mutation-driven 15-d-PGJ2 production activates NRF2 signaling to promote SG assembly [93,121,125] and cellular adaptation to chemotherapeutic stress, while SG-associated USP10 enhances antioxidant capacity to reduce ROS levels [24]. SG-mediated RNA regulation further contributes to resistance, including G3BP2-dependent sequestration of PDIA3 mRNA, which leads to DKK1 upregulation and suppression of hENT1-mediated drug uptake [124]. In parallel, recruitment of RACK1 and TRAF2 into SGs suppresses TNF signaling and apoptosis [126]. In addition, G2 phase arrest promotes SG formation through a cPLA2-Caspase-3 regulatory circuit, further enhancing resistance to chemotherapy [123]. SGs-mediated coordination of redox regulation, RNA metabolism, apoptosis suppression, and drug transport collectively drives chemoresistance in PDAC. Created with BioRender by Li, X. (2026). https://BioRender.com/zl22qfr (accessed on 30 March 2026).

SGs have emerged as critical contributors to drug resistance in PDAC (Figure 7). SGs represent another important subcellular structure involved in cellular stress responses. Increasing evidence suggests that stress granule dynamics are closely interconnected with other subcellular structure and regulatory pathways previously discussed, including oxidative stress responses, RNA metabolism, and selective autophagy-mediated protein quality control. SGs function as dynamic, non-membranous assemblies that integrate signals from multiple subcellular structures to coordinate adaptive stress responses and promote chemoresistance in PDAC.

SGs interact intimately with mitochondria, where SG-associated proteins such as ubiquitin-specific protease 10 (USP10) exert antioxidant activity, reducing ROS levels and thereby protecting mitochondria from oxidative damage [24]. This links SG dynamics directly to mitochondrial redox homeostasis. SG formation is also intimately connected to ER stress through the PERK-eIF2α pathway, a central branch of the UPR; PERK activation not only induces SGs but also regulates mitochondrial function via downstream signals [118,119]. Thus, SGs serve as a cytoplasmic extension of the ER stress response, influencing mitochondrial metabolism. Furthermore, SGs are cleared through autophagy, where autophagy receptors such as p62/SQSTM1 and CCT2 recognize SG components and facilitate their delivery to lysosomes for degradation [120]. Impairment of autophagy leads to persistence of pathological SGs, which may sequester pro-survival factors and link SG turnover to lysosomal function.

KRAS mutations play a core role in driving SG-mediated resistance. Studies have shown that gemcitabine alone does not induce SG formation. However, KRAS-mutant PDAC cells promote SG assembly by upregulating the signaling lipid 15-deoxy-delta-prostaglandin-J2 (15-d-PGJ2), which activates NRF2-mediated antioxidant pathways critical for gemcitabine resistance [93,121,122]. Given that NRF2 is a central regulator of oxidative stress responses and metabolic adaptation, its activation may coordinate SG formation with broader cellular stress-response networks in PDAC cells. Consistently, NRF2 knockdown inhibits SG formation in KRAS-mutant PDAC cells and enhances the therapeutic efficacy of gemcitabine, suggesting a potential link between SGs, NRF2, and gemcitabine resistance. Similarly, SG formation also exhibits cell cycle-dependent heterogeneity, with assembly specifically increased during G2 phase due to impaired caspase-3-mediated cleavage of cytosolic phospholipase A2 (cPLA2), promoting accumulation of the SG-inducing lipid 15-d-PGJ2 [123]. This G2 phase-specific mechanism enhances resistance to DNA-damaging agents that induce G2 arrest. In addition, 15-d-PGJ2 secreted by these cells can also act as a paracrine signal, inducing the formation of SGs and stress resistance in surrounding KRAS wild-type cancer cells, thus establishing a “non-cell autonomous” and broadly resistant protective microenvironment within the tumor [122].

SGs directly mediate drug resistance through their internal specific molecular recruitment and regulation mechanisms. For instance, the SG core component Ras GTPase-activating protein-binding protein 2 (G3BP2) recruits mRNA encoding protein disulfide isomerase family A member 3 (PDIA3) into SGs, stabilizing the transcript while repressing its translation, which leads to upregulation of Dickkopf Wnt signaling pathway inhibitor 1 (DKK1). Elevated DKK1 expression diminishes cellular uptake of gemcitabine by downregulating hENT expression, thereby promoting chemoresistance in PDAC [124].

In conclusion, SGs are key contributors to chemoresistance in PDAC. The mechanisms by which SGs promote chemoresistance include their interaction with mitochondria, ER, lysosomes, and the cell cycle machinery, SGs act as a dynamic regulatory hub that integrates organellar stress signals, enabling PDAC cells to adapt to and survive therapeutic pressure. This framework provides a deeper understanding of the complex roles SGs play in PDAC chemoresistance and highlights the potential of targeting SG dynamics (such as their formation, disassembly, and stress response regulation) as a novel strategy to overcome drug resistance in PDAC.

## 9. Crosstalk Among Subcellular Structures in Pancreatic Cancer Drug Resistance

**Figure 8:**
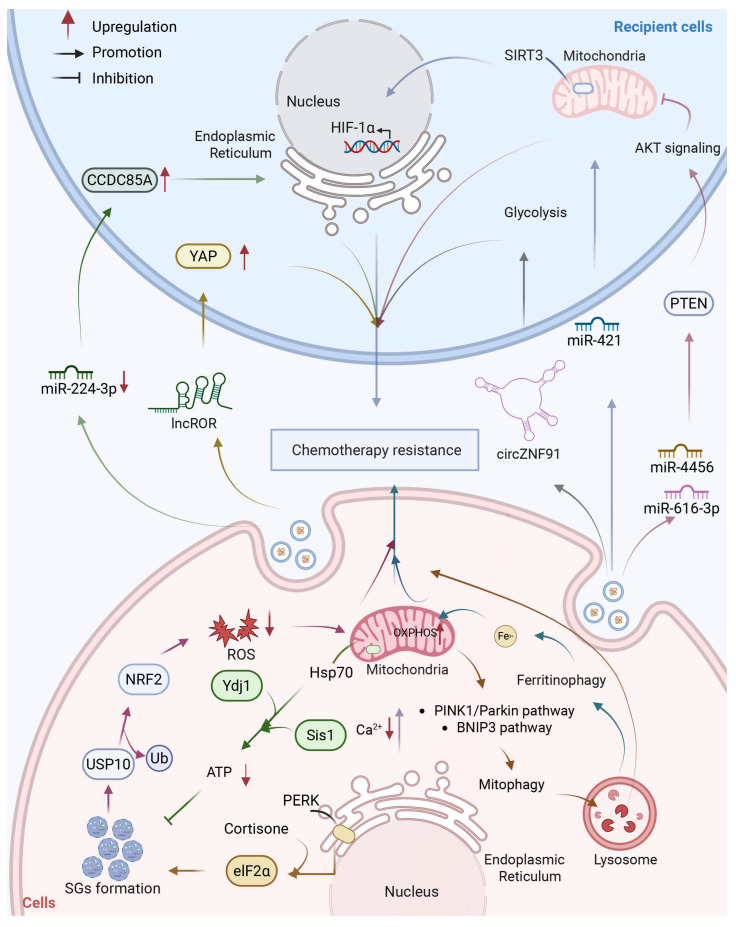
Integrative network of subcellular structural homeostasis in PDAC drug resistance. Mitochondria function as a central hub integrating signals from the ER, lysosomes, and stress granules to regulate Ca^2+^ transfer (mediated by the GRP75–IP_3_R–VDAC1 complex), mitophagy, redox balance, and metabolic reprogramming (e.g., OXPHOS and glycolysis) [24,32,39,79,118,119,120,127,128,129,130,131]. In parallel, exosomes deliver regulatory cargos (miR-421, miR-4456, miR-616-3p, miR-224-3p, circZNF91, and lncROR) to recipient cells, modulating mitochondrial function (SIRT3/HIF-1α), AKT signaling (via PTEN), and ER stress (PERK pathway) [52,104,105,106,107,108]. These coordinated intra- and intercellular mechanisms drive metabolic adaptation and promote chemoresistance in PDAC. Created with BioRender by Li, X. (2026). https://BioRender.com/v48uw98 (accessed on 30 March 2026).

The research on the contribution of single subcellular structures to the PDAC resistance through regulating homeostasis has been widely clarified. However, these structures do not operate in isolation. Rather, they form an interconnected network, where dynamic communication between organelles enables PDAC cells to mount a coordinated, multifaceted adaptive response to therapeutic stress. This section synthesizes the key nodes of inter-organelle crosstalk, with a focus on mitochondria as the central intracellular signaling hub and exosomes as critical intercellular messengers that propagate resistant phenotypes to recipient cells. By integrating findings already discussed, we aim to summarize how the drug resistance network in PDAC is shaped from the perspective of the functional interaction between subcellular structures (Figure 8).

### 9.1. Mitochondria as the Intracellular Signaling Hub

Mitochondria occupy a central position in the cellular stress response network, receiving and integrating signals from multiple organelles and translating them into adaptive outputs that determine cell fate under therapeutic pressure. This hub function is achieved through three major axes of inter-organelle communication.

Mitochondria-ER: The physical and functional interface between mitochondria and the ER, known as mitochondria-associated membranes (MAMs), represents a critical node for Ca^2+^ signaling and metabolic coordination. The GRP75-IP_3_R-VDAC1 complex forms a high-conductance Ca^2+^ transfer tunnel at MAMs, allowing ER-released Ca^2+^ to be rapidly taken up by mitochondria. This Ca^2+^ transfer serves a dual purpose: under physiological conditions, it stimulates OXPHOS and ATP production to fuel cellular functions; under pathological stress, sustained Ca^2+^ transfer can trigger mitochondrial Ca^2+^ overload, opening the mitochondrial permeability transition pore (mPTP) and compromising ATP synthesis. In PDAC, oncogenic KRAS mutations disrupt MAM integrity, impairing GRP75-IP_3_R-VDAC1 complex function and reducing mitochondrial Ca^2+^ uptake. This forces cancer cells to rely on glycolysis for ATP production, creating a vicious cycle of cytosolic Ca^2+^ overload and metabolic dysfunction that paradoxically promotes survival under therapeutic stress [32,39]. Furthermore, ER stress signals propagated through MAMs activate mitochondrial adaptive responses, including metabolic reprogramming and redox balancing, which collectively enhance chemoresistance.

Mitochondria-Lysosome: Mitochondria and lysosomes engage in bidirectional communication essential for quality control and metabolic adaptation. Mitophagy is mediated by PINK1/Parkin-dependent and BNIP3-dependent pathways, delivering dysfunctional mitochondria to lysosomes for degradation. This process maintains a healthy mitochondrial pool and prevents the release of pro-apoptotic factors, thereby promoting cell survival under chemotherapeutic stress [127,128]. Conversely, lysosomes supply mitochondria with essential metabolites through ferritinophagy. Lysosomal degradation of ferritin releases iron, which is transported to mitochondria to support OXPHOS and Fe-S cluster biogenesis, sustaining mitochondrial function and chemoresistance [79,129]. This lysosome-mitochondria iron shuttle exemplifies how organellar crosstalk directly couples nutrient recycling to bioenergetic adaptation.

Mitochondria-SGs: SGs and mitochondria engage in reciprocal communication that integrates oxidative stress responses with energy metabolism. SG-associated proteins such as USP10 exert antioxidant activity, reducing ROS levels and thereby protecting mitochondria from oxidative damage [24]. This links SG dynamics directly to mitochondrial redox homeostasis. Furthermore, SG formation is intimately connected to ER stress through the PERK-eIF2α pathway, which not only induces SGs but also regulates mitochondrial function via downstream signals [118,119,130]. Through this axis, ER stress signals are relayed to both SGs and mitochondria, coordinating a unified adaptive response. Conversely, mitochondrial dysfunction and ATP depletion can influence SG dynamics, as autophagy-mediated SG clearance requires adequate energy supply [120,131]. This bidirectional interplay positions mitochondria and SGs as co-regulated nodes in the cellular stress response network.

### 9.2. Exosomes as Intercellular Signaling Vectors

While mitochondria integrate intracellular signals, exosomes serve as intercellular messengers that disseminate adaptive states to neighboring and distant cells. Exosomes carry a diverse cargo of proteins, lipids, and nucleic acids that reflect the physiological state of donor cells. Upon uptake by recipient cells, these exosomal cargos can profoundly reprogram the function of multiple organelles, thereby propagating drug-resistant phenotypes throughout the tumor population. Critically, this intercellular communication extends the organellar crosstalk network beyond the boundaries of a single cell.

Exosome-Mitochondria (recipient cells): Exosomes derived from CAFs carry miR-421, which targets the mitochondrial deacetylase SIRT3 in recipient PDAC cells. SIRT3 downregulation leads to mitochondrial dysfunction and activates HIF-1α-mediated transcriptional reprogramming, promoting glycolysis and survival under hypoxic conditions [106,107]. Similarly, exosomes from pancreatic stellate cells (PSCs) deliver miR-4456 and miR-616-3p, which activate AKT signaling by targeting PTEN, indirectly suppressing mitochondria-mediated apoptosis [108]. Exosomes also contribute to metabolic and phenotypic reprogramming of recipient cells under stress conditions such as hypoxia. Exosomes secreted by hypoxic PDAC cells transfer circZNF91 and lncROR to normal cells [104,105]. These examples illustrate how exosomal cargo can remotely modulate mitochondrial function, linking the stromal microenvironment to cancer cell metabolism.

Exosome-ER (recipient cells): Exosomes secreted by normal fibroblasts carry miR-224-3p, which targets CCDC85A and modulates PERK-mediated ER stress responses. Upon conversion of normal fibroblasts to CAFs, miR-224-3p downregulation relieves CCDC85A suppression, rendering cancer cells resistant to ER stress-induced apoptosis [52]. This mechanism directly links exosomal signaling to the ER stress response network in recipient cells, demonstrating how intercellular communication can reprogram organellar stress adaptation.

## 10. Discussion

PDAC drug resistance remains a formidable clinical challenge, driven by complex mechanisms that extend beyond genetic mutations and the TME. This review synthesizes the current knowledge from the perspective of subcellular structural homeostasis, and emphasizes how cell membranes, mitochondria, ER, ribosomes, lysosomes, exosomes and SGs can be functionally shaped to promote chemical resistance. Collectively, these organelles do not operate in isolation; rather, they form an integrated, dynamic network in which inter-organelle communication amplifies and stabilizes the resistant phenotype.

Mitochondria function as the central intracellular signaling hub, integrating Ca^2+^ signals from the ER (via mitochondria-associated membranes, MAMs), quality control inputs from lysosomes (through mitophagy and ferritinophagy), and oxidative stress signals from SGs. This integration enables PDAC cells to coordinate metabolic adaptation, redox balance, and survival decisions under therapeutic pressure. Simultaneously, exosomes act as intercellular signaling vectors, propagating adaptive states from donor cells to recipient cells by delivering specific miRNAs, proteins, and lipids that reprogram mitochondrial function, ER stress responses, and ferroptosis sensitivity. This exosome-mediated communication extends the organellar crosstalk network beyond the boundaries of a single cell, creating a “field effect” that disseminates resistance throughout the tumor population. The interconnected nature of this network explains why targeting single structures often fails: cancer cells can rewire their organellar interactions to bypass the blockade.

A growing repertoire of agents targeting subcellular structural homeostasis have advanced into preclinical studies or clinical trials for PDAC and other solid malignancies, highlighting the substantial translational potential of this therapeutic paradigm. In preclinical investigations, leflunomide, an agent originally approved for the treatment of arthritis, has been repurposed as an agonist of Mfn2 to drive mitochondrial fusion, with robust anti-tumor efficacy demonstrated in preclinical models of PDAC (NCT06454383). Among agents that have entered clinical evaluation, those directed at mitochondrial function are at the forefront. Phenformin, a mitochondrial complex I inhibitor, is being investigated in combination with gemcitabine for the treatment of PDAC tumors harboring a high OXPHOS transcriptional signature [132,133]. Devimistat (CPI-613), a dual inhibitor of pyruvate dehydrogenase and α-ketoglutarate dehydrogenase, failed to meet its primary efficacy endpoint in a phase III trial for metastatic PDAC, yet exhibited an acceptable safety profile (NCT03374852). Devimistat has demonstrated promising anti-tumor efficacy in numerous preclinical studies [134,135,136,137,138]. However, its failure in this phase III trial highlights the need to explore multi-target treatment strategies. Preclinical evidence indicates that devimistat-induced mitochondrial dysfunction can trigger adaptive responses in other subcellular structures. These include lysosome-dependent mitophagy to clear damaged mitochondria, enhanced peroxisomal fatty acid oxidation to bypass TCA cycle inhibition, and endoplasmic reticulum stress-mediated remodeling of the tumor secretome [139,140]. While it remains to be confirmed whether these mechanisms contributed to the clinical trial failure, it is at least partly due to the crosstalk activation between subcellular structures. Agents targeting ER homeostasis have also shown promise. ursolic acid, a modulator of ER stress, has demonstrated synergistic anti-tumor efficacy with gemcitabine in preclinical PDAC models [57]. For lysosome-directed therapy, hydroxychloroquine, which inhibits autophagy by blocking lysosomal acidification, has been evaluated in combination with MEK inhibitors (e.g., trametinib) across multiple phase I/II clinical trials, with relevant studies including, which assesses a MEK inhibitor-based regimen incorporating hydroxychloroquine for advanced PDAC harboring KRAS^G12R^ mutations (NCT05630989), and another that investigates hydroxychloroquine combined with nivolumab, nab-paclitaxel, and gemcitabine for metastatic PDAC (NCT04787991). Exosome-based therapeutic strategies have also entered clinical testing. The engineered exosome preparation iExoKras^G12D^, which delivers small interfering RNA (siRNA) specific for the oncogenic KRAS^G12D^ variant, was evaluated in a phase I trial (NCT03608631), where it demonstrated a favorable tolerability profile, with on-target engagement observed in treated patients. Separately, the calcium channel blocker amlodipine was identified in preclinical analyses as an agent with the potential to augment the anti-tumor activity of gemcitabine [141]. Collectively, these preclinical and clinical studies underscore a critical unmet need for biomarker-driven patient stratification to maximize the clinical benefit of these subcellular-targeted therapeutic approaches in PDAC.

Despite significant progress, critical gaps remain. First, the molecular mechanisms governing inter-organelle communication are incompletely understood. For instance, how ER stress signals are quantitatively transmitted through MAMs to modulate mitochondrial metabolism, or how exosomal cargo selectivity is determined, requires further investigation using advanced techniques such as proximity labeling proteomics and super-resolution microscopy. Second, PDAC exhibits profound intratumoral heterogeneity, and the functional states of subcellular structures likely differ between cancer stem cells and differentiated tumor cells, which is an unexplored area with therapeutic implications. Third, the development of non-invasive biomarkers reflecting subcellular structural homeostasis (e.g., circulating exosomal miRNAs, mitochondrial DNA mutations) could enable real-time monitoring of resistance evolution and guide treatment decisions. Fourth, combination strategies targeting multiple nodes of the organellar network, such as simultaneous inhibition of MAM integrity, mitophagy flux, and exosome biogenesis, may prove more effective than single-structure targeting. Preclinical studies should prioritize testing such rational combinations in patient-derived xenograft and organoid models. Fifth, poor tissue permeation and off-target effects present significant challenges for the clinical translation of subcellular targeted therapies. Therefore, it will be essential to develop intelligent conveying systems, such as ligand-modified nanocarriers targeting cell membrane markers or subcellular positioning signals.

## 11. Conclusions

In summary, the functional remodeling and coordinated adaptation of subcellular structures constitute a central mechanism driving drug resistance in PDAC. This framework shifts the focus from individual molecules to the complex interactions between organelles, with mitochondria as the central intracellular hub and exosomes as intercellular messengers. This approach opens new avenues for biomarker discovery and multi-target therapeutic strategies. Further mechanistic studies, coupled with innovative clinical trial designs, will pave the way for personalized treatments that disrupt the survival networks of cancer cells and ultimately improve outcomes for patients with this devastating disease.

## Data Availability

No new data were created or analyzed in this study.

## Abbreviations

The following abbreviations are used in this manuscript:

15-d-PGJ2	15-deoxy-delta-prostaglandin-J2
ABC	ATP-binding cassette
ABCB1	ATP-binding cassette subfamily B member 1
ABCB11	ATP-binding cassette subfamily B member 11
ABCB4	ATP-binding cassette subfamily B member 4
ABCC1	ATP-binding cassette subfamily C member 1
ABCC10	ATP-binding cassette subfamily C member 10
ABCC3	ATP-binding cassette subfamily C member 3
ABCC5	ATP-binding cassette subfamily C member 5
ABCG2	ATP-binding cassette subfamily G member 2
ACSL4	Acyl-CoA synthetase long chain family member 4
AGR2	Anterior gradient 2
AKT	Serine-threonine kinase
ATF4	Activating transcription factor 4
ATF6	Activating transcription factor 6
BCL2	B-cell lymphoma 2
BZW1	Basic leucine zipper and W2 domains 1
CAFs	Cancer-associated fibroblasts
CALR	Calreticulin
CAT	Catalase
CCDC85A	Coiled-coil domain containing 85A
CDA	Cytidine deaminase
CDK4/6	Cyclin-dependent kinase 4/6
CDKN2A	Cyclin dependent kinase inhibitor 2A
circRNAs	Circular RNAs
CLPTM1L	Cleft lip and palate transmembrane protein 1-like
c-Myc	Cellular myelocytomatosis oncogene
COPS6	COP9 signalosome subunit 6
COX-2	Cyclooxygenase-2
cPLA2	Cytosolic phospholipase A2
CUGBP2	CUG triplet repeat, RNA binding protein 2
CYP51A1	Cytochrome P450 family 51 subfamily A member 1
DCAF1	DDB1 and CUL4 associated factor 1
dCK	Deoxycytidine kinase
dFdCDP	Gemcitabine diphosphate
dFdCTP	Gemcitabine triphosphate
DKK1	Dickkopf Wnt signaling pathway inhibitor 1
Drp1	Dynamin-related protein 1
EGFR	Epidermal growth factor receptor
eIF2α	Eukaryotic translation initiation factor 2 alpha
EMT	Epithelial–mesenchymal transition
ENT1	Equilibrative nucleoside transporter 1
ER	Endoplasmic reticulum
ERK	Extracellular signal-regulated kinase
ERK2	Extracellular signal-regulated kinase 2
ERN1	Endoplasmic reticulum to nucleus signaling 1
EVs	Extracellular vesicles
FBW7	F-box and WD repeat domain containing 7
FOLFIRINOX	FOLFIRINOX (comprising 5-fluorouracil, leucovorin, irinotecan, and oxaliplatin)
G3BP2	Ras GTPase-activating protein-binding protein 2
GAA	Acid α-glucosidase
GDMCN2	Gemcitabine-Derived Multifunctional Carbon Nitride Nanocage 2
GRP78	Glucose-regulated protein 78kDa
hCNTs	Human concentrative nucleoside transporters
hENT1	Human equilibrative nucleoside transporter 1
hENTs	Human equilibrative nucleoside transporters
HIF-1α	Hypoxia-inducible factor 1 alpha
HIF-2α	Hypoxia-inducible factor 2 alpha
HO-1	Heme oxygenase 1
HSP47	Heat shock protein 47
IAPs	Inhibitor of apoptosis proteins
IGF2BP1	Insulin-like growth factor 2 mRNA binding protein 1
IGF2BP2	Insulin-like growth factor 2 mRNA binding protein 2
IRE1	Inositol-requiring enzyme 1
IRE1α	Inositol-requiring enzyme 1 alpha
IRES	Internal ribosomal entry site
lncRNA	Long non-coding RNA
MAPK	Mitogen-activated protein kinase
MDR1	Multidrug resistance protein 1
MEK	Mitogen-activated protein kinase kinase
Mfn2	Mitofusin 2
MHC-I	Major histocompatibility complex class I
MIA2	Melanoma inhibitory activity 2
MIF	Macrophage migration inhibitory factor
miRNAs	MicroRNAs
MQC	Mitochondrial quality control
mTORC1	Mechanistic target of rapamycin kinase complex 1
Nab-paclitaxel	Nanoparticle albumin-bound paclitaxel
NAF-1	Nutrient deprivation autophagy factor-1
NBR1	Next to BRCA1 gene 1
NCOA4	Nuclear receptor coactivator 4
NDPK	Nucleoside diphosphate kinase
NDRG1	N-myc downstream regulated 1
NFAT	Nuclear factor of activated T-cells
NMPK	Nucleoside monophosphate kinase
NPM1	Nucleophosmin 1
NRF2	Nuclear factor erythroid 2-related factor 2
P2RX7	Purinergic receptor P2X7
PDAC	Pancreatic ductal adenocarcinoma
PDGFA	Platelet-derived growth factor subunit A
PDIA3	Protein disulfide isomerase family A member 3
PERK	Protein kinase R-like ER kinase
PGM3	Phosphoglucomutase 3
P-gp	P-glycoprotein
PI3Kα	Phosphoinositide 3-kinase alpha
Pin1	Peptidylprolyl cis/trans isomerase, NIMA-interacting 1
PKR	Protein kinase R
PPP5C	Protein phosphatase 5 catalytic subunit
PTEN	Phosphatase and tensin homolog
RACK1	Receptor for activated c kinase 1
RAGE	Receptor for advanced glycosylation end-products
RIPK2	Receptor-interacting protein kinase 2
ROS	Reactive oxygen species
RRM1/2	Ribonucleotide reductase catalytic subunit M1/2
RRP9	Ribosomal RNA processing 9
RUNX1	RUNX family transcription factor 1
SGs	Stress granules
SLC1A5	Solute carrier family 1 member 5
SLC7A11	Solute carrier family 7 member 11
SMAC	Second mitochondria-derived activator of caspases
SMAD4	Mothers against decapentaplegic homolog 4
SNAIL	Snail family transcriptional repressor 1
SOCE	Store-operated Ca^2+^ entry
SOD2	Superoxide dismutase 2
SP1	Specificity protein 1
STIM1	Stromal interaction molecule 1
TFEB	Transcription factor EB
TGFβ	Transforming growth factor beta
TGM2	Transglutaminase 2
TIA-1	T-cell intracellular antigen 1
TME	Tumor microenvironment
TMEM175	Transmembrane protein 175
TMOD3	Tropomodulin 3
TP53	Tumor protein p53
TP53INP1	Tumor protein p53 inducible nuclear protein 1
TRAF2	TNF receptor-associated factor 2
UPR	Unfolded protein response
USP10	Ubiquitin-specific protease 10
USP7	Ubiquitin-specific protease 7
YAP	Yes-associated protein

## References

[1] Luo G., Fan Z., Gong Y., Jin K., Yang C., Cheng H., Huang D., Ni Q., Liu C., Yu X. (2019). Characteristics and Outcomes of Pancreatic Cancer by Histological Subtypes. Pancreas.

[2] Zhou C., Dong X., Li S., Xi Y., Liu Y., Qian X., Song Z., Zhou L., Zhang R., Lyu H. (2025). Serine/threonine/tyrosine kinase 1 drives pancreatic carcinogenesis via GSK3beta sequestration-mediated Wnt/beta-catenin pathway hyperactivation. Signal Transduct. Target. Ther..

[3] Siegel R.L., Giaquinto A.N., Jemal A. (2024). Cancer statistics, 2024. CA Cancer J. Clin..

[4] Fuchs C.S., Colditz G.A., Stampfer M.J., Giovannucci E.L., Hunter D.J., Rimm E.B., Willett W.C., Speizer F.E. (1996). A prospective study of cigarette smoking and the risk of pancreatic cancer. Arch. Intern. Med..

[5] Park W., Chawla A., O’Reilly E.M. (2021). Pancreatic Cancer: A Review. JAMA.

[6] Huxley R., Ansary-Moghaddam A., Berrington de Gonzalez A., Barzi F., Woodward M. (2005). Type-II diabetes and pancreatic cancer: A meta-analysis of 36 studies. Br. J. Cancer.

[7] Halbrook C.J., Lyssiotis C.A., Pasca di Magliano M., Maitra A. (2023). Pancreatic cancer: Advances and challenges. Cell.

[8] Shah A., Ganguly K., Rauth S., Sheree S.S., Khan I., Ganti A.K., Ponnusamy M.P., Kumar S., Jain M., Batra S.K. (2024). Unveiling the resistance to therapies in pancreatic ductal adenocarcinoma. Drug Resist. Updates.

[9] Adamska A., Domenichini A., Falasca M. (2017). Pancreatic Ductal Adenocarcinoma: Current and Evolving Therapies. Int. J. Mol. Sci..

[10] Von Hoff D.D., Ervin T., Arena F.P., Chiorean E.G., Infante J., Moore M., Seay T., Tjulandin S.A., Ma W.W., Saleh M.N. (2013). Increased survival in pancreatic cancer with nab-paclitaxel plus gemcitabine. N. Engl. J. Med..

[11] Stoop T.F., Javed A.A., Oba A., Koerkamp B.G., Seufferlein T., Wilmink J.W., Besselink M.G. (2025). Pancreatic cancer. Lancet.

[12] Conroy T., Desseigne F., Ychou M., Bouche O., Guimbaud R., Becouarn Y., Adenis A., Raoul J.L., Gourgou-Bourgade S., de la Fouchardiere C. (2011). FOLFIRINOX versus gemcitabine for metastatic pancreatic cancer. N. Engl. J. Med..

[13] Lyu H., Kong J., Chen J., Zhang R., Xiao S., Guo D., Zhang Q., Chen X.Z., Tang J., Zhou C. (2024). The Emerging Scenario of Ferroptosis in Pancreatic Cancer Tumorigenesis and Treatment. Int. J. Mol. Sci..

[14] Ezrova Z., Nahacka Z., Stursa J., Werner L., Vlcak E., Kralova Viziova P., Berridge M.V., Sedlacek R., Zobalova R., Rohlena J. (2021). SMAD4 loss limits the vulnerability of pancreatic cancer cells to complex I inhibition via promotion of mitophagy. Oncogene.

[15] Ghiglione N., Abbo D., Bushunova A., Costamagna A., Porporato P.E., Martini M. (2025). Metabolic plasticity in pancreatic cancer: The mitochondrial connection. Mol. Metab..

[16] Xie L.L., Shi F., Tan Z., Li Y., Bode A.M., Cao Y. (2018). Mitochondrial network structure homeostasis and cell death. Cancer Sci..

[17] Kashatus J.A., Nascimento A., Myers L.J., Sher A., Byrne F.L., Hoehn K.L., Counter C.M., Kashatus D.F. (2015). Erk2 phosphorylation of Drp1 promotes mitochondrial fission and MAPK-driven tumor growth. Mol. Cell.

[18] Ge J., Ge C. (2019). Rab14 overexpression regulates gemcitabine sensitivity through regulation of Bcl-2 and mitochondrial function in pancreatic cancer. Virchows Arch..

[19] Clarke W.R., Amundadottir L., James M.A. (2019). CLPTM1L/CRR9 ectodomain interaction with GRP78 at the cell surface signals for survival and chemoresistance upon ER stress in pancreatic adenocarcinoma cells. Int. J. Cancer.

[20] Schwarz D.S., Blower M.D. (2016). The endoplasmic reticulum: Structure, function and response to cellular signaling. Cell Mol. Life Sci..

[21] She C., Wu C., Guo W., Xie Y., Li S., Liu W., Xu C., Li H., Cao P., Yang Y. (2023). Combination of RUNX1 inhibitor and gemcitabine mitigates chemo-resistance in pancreatic ductal adenocarcinoma by modulating BiP/PERK/eIF2alpha-axis-mediated endoplasmic reticulum stress. J. Exp. Clin. Cancer Res..

[22] Grout J.A., Sirven P., Leader A.M., Maskey S., Hector E., Puisieux I., Steffan F., Cheng E., Tung N., Maurin M. (2022). Spatial Positioning and Matrix Programs of Cancer-Associated Fibroblasts Promote T-cell Exclusion in Human Lung Tumors. Cancer Discov..

[23] Xu H., Xue S., Sun Y., Ma J., Li S., Wang Y., Mao T., Ge W., Yue M., Shentu D. (2025). CREB3L1 facilitates pancreatic tumor progression and reprograms intratumoral tumor-associated macrophages to shape an immunotherapy-resistance microenvironment. J. Immunother. Cancer.

[24] Takahashi M., Higuchi M., Matsuki H., Yoshita M., Ohsawa T., Oie M., Fujii M. (2013). Stress granules inhibit apoptosis by reducing reactive oxygen species production. Mol. Cell Biol..

[25] Wang J., Fan P., Shen P., Fan C., Zhao P., Yao S., Dong K., Ling R., Chen S., Zhang J. (2024). XBP1s activates METTL3/METTL14 for ER-phagy and paclitaxel sensitivity regulation in breast cancer. Cancer Lett..

[26] Mini E., Nobili S., Caciagli B., Landini I., Mazzei T. (2006). Cellular pharmacology of gemcitabine. Ann. Oncol..

[27] Zeng S., Pottler M., Lan B., Grutzmann R., Pilarsky C., Yang H. (2019). Chemoresistance in Pancreatic Cancer. Int. J. Mol. Sci..

[28] Mohelnikova-Duchonova B., Brynychova V., Oliverius M., Honsova E., Kala Z., Muckova K., Soucek P. (2013). Differences in transcript levels of ABC transporters between pancreatic adenocarcinoma and nonneoplastic tissues. Pancreas.

[29] Pote M.S., Gacche R.N. (2023). ATP-binding cassette efflux transporters and MDR in cancer. Drug Discov. Today.

[30] Gu J., Huang W., Wang X., Zhang J., Tao T., Zheng Y., Liu S., Yang J., Chen Z.S., Cai C.Y. (2022). Hsa-miR-3178/RhoB/PI3K/Akt, a novel signaling pathway regulates ABC transporters to reverse gemcitabine resistance in pancreatic cancer. Mol. Cancer.

[31] Bergman A.M., Pinedo H.M., Talianidis I., Veerman G., Loves W.J., van der Wilt C.L., Peters G.J. (2003). Increased sensitivity to gemcitabine of P-glycoprotein and multidrug resistance-associated protein-overexpressing human cancer cell lines. Br. J. Cancer.

[32] Petersen O.H., Gerasimenko J.V., Gerasimenko O.V., Gryshchenko O., Peng S. (2021). The roles of calcium and ATP in the physiology and pathology of the exocrine pancreas. Physiol. Rev..

[33] Salih M., Petersen O.H. (2026). Activation of pancreatic acinar cells by very low concentrations of cholecystokinin: Mechanism and implications for physiology and pathology. Pancreatology.

[34] Chan D.C. (2020). Mitochondrial Dynamics and Its Involvement in Disease. Annu. Rev. Pathol..

[35] Chen H., Chan D.C. (2005). Emerging functions of mammalian mitochondrial fusion and fission. Hum. Mol. Genet..

[36] Du C., Fang M., Li Y., Li L., Wang X. (2000). Smac, a mitochondrial protein that promotes cytochrome c-dependent caspase activation by eliminating IAP inhibition. Cell.

[37] Hashim Y.M., Vangveravong S., Sankpal N.V., Binder P.S., Liu J., Goedegebuure S.P., Mach R.H., Spitzer D., Hawkins W.G. (2017). The Targeted SMAC Mimetic SW IV-134 is a strong enhancer of standard chemotherapy in pancreatic cancer. J. Exp. Clin. Cancer Res..

[38] Hashim Y.M., Spitzer D., Vangveravong S., Hornick M.C., Garg G., Hornick J.R., Goedegebuure P., Mach R.H., Hawkins W.G. (2014). Targeted pancreatic cancer therapy with the small molecule drug conjugate SW IV-134. Mol. Oncol..

[39] Zhang T., Chen S., Li L., Jin Y., Liu S., Liu Z., Shi F., Xie L., Guo P., Cannon A.C. (2024). PFKFB3 controls acinar IP3R-mediated Ca^2+^ overload to regulate acute pancreatitis severity. JCI Insight.

[40] Cheng L., Yan B., Chen K., Jiang Z., Zhou C., Cao J., Qian W., Li J., Sun L., Ma J. (2018). Resveratrol-Induced Downregulation of NAF-1 Enhances the Sensitivity of Pancreatic Cancer Cells to Gemcitabine via the ROS/Nrf2 Signaling Pathways. Oxid. Med. Cell Longev..

[41] Mukhopadhyay S., Goswami D., Adiseshaiah P.P., Burgan W., Yi M., Guerin T.M., Kozlov S.V., Nissley D.V., McCormick F. (2020). Undermining Glutaminolysis Bolsters Chemotherapy While NRF2 Promotes Chemoresistance in KRAS-Driven Pancreatic Cancers. Cancer Res..

[42] Yoo H.C., Park S.J., Nam M., Kang J., Kim K., Yeo J.H., Kim J.K., Heo Y., Lee H.S., Lee M.Y. (2020). A Variant of SLC1A5 Is a Mitochondrial Glutamine Transporter for Metabolic Reprogramming in Cancer Cells. Cell Metab..

[43] Yu M., Nguyen N.D., Huang Y., Lin D., Fujimoto T.N., Molkentine J.M., Deorukhkar A., Kang Y., San Lucas F.A., Fernandes C.J. (2019). Mitochondrial fusion exploits a therapeutic vulnerability of pancreatic cancer. JCI Insight.

[44] Ye K., Zhou S., Gong X., Zhu Z., Xiao M., Liang S. (2025). TGM2-P2RX7 loop promotes gemcitabine resistance in pancreatic cancer by modulating glutamine metabolism and mitophagy. Cell Death Discov..

[45] Zhao C., He R., Shen M., Zhu F., Wang M., Liu Y., Chen H., Li X., Qin R. (2019). PINK1/Parkin-Mediated Mitophagy Regulation by Reactive Oxygen Species Alleviates Rocaglamide A-Induced Apoptosis in Pancreatic Cancer Cells. Front. Pharmacol..

[46] Yule D.I., Takano T. (2024). Pacing intracellular Ca(^2+^) signals in exocrine acinar cells. J. Physiol..

[47] Oakes S.A., Papa F.R. (2015). The role of endoplasmic reticulum stress in human pathology. Annu. Rev. Pathol..

[48] Garcia-Carbonero N., Li W., Cabeza-Morales M., Martinez-Useros J., Garcia-Foncillas J. (2018). New Hope for Pancreatic Ductal Adenocarcinoma Treatment Targeting Endoplasmic Reticulum Stress Response: A Systematic Review. Int. J. Mol. Sci..

[49] Chen X., Cubillos-Ruiz J.R. (2021). Endoplasmic reticulum stress signals in the tumour and its microenvironment. Nat. Rev. Cancer.

[50] Celik C., Lee S.Y.T., Yap W.S., Thibault G. (2023). Endoplasmic reticulum stress and lipids in health and diseases. Prog. Lipid Res..

[51] Yoneda A., Minomi K., Tamura Y. (2021). Heat shock protein 47 confers chemoresistance on pancreatic cancer cells by interacting with calreticulin and IRE1alpha. Cancer Sci..

[52] Takahashi S., Takagane K., Itoh G., Kuriyama S., Umakoshi M., Goto A., Yanagihara K., Yashiro M., Iijima K., Tanaka M. (2023). CCDC85A is regulated by miR-224-3p and augments cancer cell resistance to endoplasmic reticulum stress. Front. Oncol..

[53] Hong X., Li Z.X., Hou J., Zhang H.Y., Zhang C.Y., Zhang J., Sun H., Pang L.H., Wang T., Deng Z.H. (2021). Effects of ER-resident and secreted AGR2 on cell proliferation, migration, invasion, and survival in PANC-1 pancreatic cancer cells. BMC Cancer.

[54] Gao Z., Janakiraman H., Xiao Y., Kang S.W., Dong J., Choi J., Ogretmen B., Lee H.S., Camp E.R. (2024). Sphingosine-1-Phosphate Inhibition Increases Endoplasmic Reticulum Stress to Enhance Oxaliplatin Sensitivity in Pancreatic Cancer. World J. Oncol..

[55] Ricciardiello F., Gang Y., Palorini R., Li Q., Giampa M., Zhao F., You L., La Ferla B., De Vitto H., Guan W. (2020). Hexosamine pathway inhibition overcomes pancreatic cancer resistance to gemcitabine through unfolded protein response and EGFR-Akt pathway modulation. Oncogene.

[56] Kutschat A.P., Hamdan F.H., Wang X., Wixom A.Q., Najafova Z., Gibhardt C.S., Kopp W., Gaedcke J., Strobel P., Ellenrieder V. (2021). STIM1 Mediates Calcium-Dependent Epigenetic Reprogramming in Pancreatic Cancer. Cancer Res..

[57] Lin J.H., Chen S.Y., Lu C.C., Lin J.A., Yen G.C. (2020). Ursolic acid promotes apoptosis, autophagy, and chemosensitivity in gemcitabine-resistant human pancreatic cancer cells. Phytother. Res..

[58] Zhao Z., Wu Y., Liang X., Liu J., Luo Y., Zhang Y., Li T., Liu C., Luo X., Chen J. (2023). Sonodynamic Therapy of NRP2 Monoclonal Antibody-Guided MOFs@COF Targeted Disruption of Mitochondrial and Endoplasmic Reticulum Homeostasis to Induce Autophagy-Dependent Ferroptosis. Adv. Sci..

[59] Kong B., Wu W., Valkovska N., Jager C., Hong X., Nitsche U., Friess H., Esposito I., Erkan M., Kleeff J. (2015). A common genetic variation of melanoma inhibitory activity-2 labels a subtype of pancreatic adenocarcinoma with high endoplasmic reticulum stress levels. Sci. Rep..

[60] Chatterjee S., Naeli P., Onar O., Simms N., Garzia A., Hackett A., Coyle K., Harris Snell P., McGirr T., Sawant T.N. (2024). Ribosome Quality Control mitigates the cytotoxicity of ribosome collisions induced by 5-Fluorouracil. Nucleic Acids Res..

[61] Li Z., Ge Y., Dong J., Wang H., Zhao T., Wang X., Liu J., Gao S., Shi L., Yang S. (2022). BZW1 Facilitates Glycolysis and Promotes Tumor Growth in Pancreatic Ductal Adenocarcinoma Through Potentiating eIF2alpha Phosphorylation. Gastroenterology.

[62] Zhang Y., Gao H., Tang A., Lyu H., Fan Z., Guo J., Wang Y., Yi H., Pan Q., Luo H. (2026). CSN6 Promotes Pancreatic Cancer Progression and Gemcitabine Resistance via Antagonizing DCAF1-Mediated Ubiquitination of NPM1. Adv. Sci. (Weinh).

[63] Zhang Z., Yu H., Yao W., Zhu N., Miao R., Liu Z., Song X., Xue C., Cai C., Cheng M. (2022). RRP9 promotes gemcitabine resistance in pancreatic cancer via activating AKT signaling pathway. Cell Commun. Signal.

[64] Iadevaia V., Liu R., Proud C.G. (2014). mTORC1 signaling controls multiple steps in ribosome biogenesis. Semin. Cell Dev. Biol..

[65] Heilmann A.M., Perera R.M., Ecker V., Nicolay B.N., Bardeesy N., Benes C.H., Dyson N.J. (2014). CDK4/6 and IGF1 receptor inhibitors synergize to suppress the growth of p16INK4A-deficient pancreatic cancers. Cancer Res..

[66] Rowell M.C., Deschenes-Simard X., Lopes-Paciencia S., Le Calve B., Kalegari P., Mignacca L., Fernandez-Ruiz A., Guillon J., Lessard F., Bourdeau V. (2023). Targeting ribosome biogenesis reinforces ERK-dependent senescence in pancreatic cancer. Cell Cycle.

[67] Jakstaite A., Maziukiene A., Silkuniene G., Kmieliute K., Dauksa A., Paskauskas S., Gulbinas A., Dambrauskas Z. (2016). Upregulation of cugbp2 increases response of pancreatic cancer cells to chemotherapy. Langenbecks Arch. Surg..

[68] Ballabio A., Bonifacino J.S. (2020). Lysosomes as dynamic regulators of cell and organismal homeostasis. Nat. Rev. Mol. Cell Biol..

[69] Lawrence R.E., Zoncu R. (2019). The lysosome as a cellular centre for signalling, metabolism and quality control. Nat. Cell Biol..

[70] Saftig P., Puertollano R. (2021). How Lysosomes Sense, Integrate, and Cope with Stress. Trends Biochem. Sci..

[71] Zhang R., Zhang X., Bai H., Cheng Q., Yao X., Li S., Torraca V., Yan C., Dong X., Miao S. (2025). DRAM1 promotes the stability of lysosomal VAMP8 to enhance autolysosome formation and facilitates the extravasation. Nat. Commun..

[72] Saftig P., Klumperman J. (2009). Lysosome biogenesis and lysosomal membrane proteins: Trafficking meets function. Nat. Rev. Mol. Cell Biol..

[73] Aits S., Jaattela M. (2013). Lysosomal cell death at a glance. J. Cell Sci..

[74] Boya P., Kroemer G. (2008). Lysosomal membrane permeabilization in cell death. Oncogene.

[75] Koikawa K., Kibe S., Suizu F., Sekino N., Kim N., Manz T.D., Pinch B.J., Akshinthala D., Verma A., Gaglia G. (2021). Targeting Pin1 renders pancreatic cancer eradicable by synergizing with immunochemotherapy. Cell.

[76] Ge F., Zhu H., Liu X., Li Y., Guo R., Zeng C., Yuan T., Yang L., Dong X., Wu Y. (2025). IGF2BP2 stabilized by USP7 promotes cancer-associated fibroblast activation and attenuates gemcitabine sensitivity in PDAC. Cell Rep..

[77] Hu Q., Qin Y., Zhang B., Liang C., Ji S., Shi S., Xu W., Xiang J., Liang D., Ni Q. (2017). FBW7 increases the chemosensitivity of pancreatic cancer cells to gemcitabine through upregulation of ENT1. Oncol. Rep..

[78] Hou W., Xie Y., Song X., Sun X., Lotze M.T., Zeh H.J., Kang R., Tang D. (2016). Autophagy promotes ferroptosis by degradation of ferritin. Autophagy.

[79] Jain V., Amaravadi R.K. (2022). Pumping Iron: Ferritinophagy Promotes Survival and Therapy Resistance in Pancreatic Cancer. Cancer Discov..

[80] He Z., Zheng D., Li F., Chen L., Wu C., Zeng Z., Yu C. (2025). TMOD3 accelerated resistance to immunotherapy in KRAS-mutated pancreatic cancer through promoting autophagy-dependent degradation of ASCL4. Drug Resist. Updat..

[81] Yamamoto K., Venida A., Yano J., Biancur D.E., Kakiuchi M., Gupta S., Sohn A.S.W., Mukhopadhyay S., Lin E.Y., Parker S.J. (2020). Autophagy promotes immune evasion of pancreatic cancer by degrading MHC-I. Nature.

[82] Sang W., Zhou Y., Chen H., Yu C., Dai L., Liu Z., Chen L., Fang Y., Ma P., Wu X. (2024). Receptor-interacting Protein Kinase 2 Is an Immunotherapy Target in Pancreatic Cancer. Cancer Discov..

[83] Zhang Z., Song B., Wei H., Liu Y., Zhang W., Yang Y., Sun B. (2024). NDRG1 overcomes resistance to immunotherapy of pancreatic ductal adenocarcinoma through inhibiting ATG9A-dependent degradation of MHC-1. Drug Resist. Updat..

[84] Chen F., Tang H., Li C., Kang R., Tang D., Liu J. (2025). CYP51A1 drives resistance to pH-dependent cell death in pancreatic cancer. Nat. Commun..

[85] Hamura R., Shirai Y., Shimada Y., Saito N., Taniai T., Horiuchi T., Takada N., Kanegae Y., Ikegami T., Ohashi T. (2021). Suppression of lysosomal acid alpha-glucosidase impacts the modulation of transcription factor EB translocation in pancreatic cancer. Cancer Sci..

[86] Marchand B., Poulin M.A., Lawson C., Tai L.H., Jean S., Boucher M.J. (2023). Gemcitabine promotes autophagy and lysosomal function through ERK- and TFEB-dependent mechanisms. Cell Death Discov..

[87] Zhao B., Dierichs L., Gu J.N., Trajkovic-Arsic M., Axel Hilger R., Savvatakis K., Vega-Rubin-de-Celis S., Liffers S.T., Pena-Llopis S., Behrens D. (2020). TFEB-mediated lysosomal biogenesis and lysosomal drug sequestration confer resistance to MEK inhibition in pancreatic cancer. Cell Death Discov..

[88] Zhang F., Jiang J., Qian H., Yan Y., Xu W. (2023). Exosomal circRNA: Emerging insights into cancer progression and clinical application potential. J. Hematol. Oncol..

[89] Creeden J.F., Sevier J., Zhang J.T., Lapitsky Y., Brunicardi F.C., Jin G., Nemunaitis J., Liu J.Y., Kalinoski A., Rao D. (2024). Smart exosomes enhance PDAC targeted therapy. J. Control. Release.

[90] Eckford P.D., Sharom F.J. (2009). ABC efflux pump-based resistance to chemotherapy drugs. Chem. Rev..

[91] Miller D.W., Fontain M., Kolar C., Lawson T. (1996). The expression of multidrug resistance-associated protein (MRP) in pancreatic adenocarcinoma cell lines. Cancer Lett..

[92] Zhang Y.K., Wang Y.J., Gupta P., Chen Z.S. (2015). Multidrug Resistance Proteins (MRPs) and Cancer Therapy. AAPS J..

[93] Kim E.H., Surh Y.J. (2006). 15-deoxy-Delta12,14-prostaglandin J2 as a potential endogenous regulator of redox-sensitive transcription factors. Biochem. Pharmacol..

[94] Patel G.K., Khan M.A., Bhardwaj A., Srivastava S.K., Zubair H., Patton M.C., Singh S., Khushman M., Singh A.P. (2017). Exosomes confer chemoresistance to pancreatic cancer cells by promoting ROS detoxification and miR-155-mediated suppression of key gemcitabine-metabolising enzyme, DCK. Br. J. Cancer.

[95] Ciccolini J., Serdjebi C., Peters G.J., Giovannetti E. (2016). Pharmacokinetics and pharmacogenetics of Gemcitabine as a mainstay in adult and pediatric oncology: An EORTC-PAMM perspective. Cancer Chemother. Pharmacol..

[96] Zhao H., Wu S., Li H., Duan Q., Zhang Z., Shen Q., Wang C., Yin T. (2019). ROS/KRAS/AMPK Signaling Contributes to Gemcitabine-Induced Stem-like Cell Properties in Pancreatic Cancer. Mol. Ther. Oncol..

[97] Richards K.E., Zeleniak A.E., Fishel M.L., Wu J., Littlepage L.E., Hill R. (2017). Cancer-associated fibroblast exosomes regulate survival and proliferation of pancreatic cancer cells. Oncogene.

[98] Richards K.E., Xiao W., Hill R., on behalf of the USC Pancreas Research Team, on behalf of the USC Pancreas Research Team (2022). Cancer-Associated Fibroblasts Confer Gemcitabine Resistance to Pancreatic Cancer Cells through PTEN-Targeting miRNAs in Exosomes. Cancers.

[99] Fang Y., Zhou W., Rong Y., Kuang T., Xu X., Wu W., Wang D., Lou W. (2019). Exosomal miRNA-106b from cancer-associated fibroblast promotes gemcitabine resistance in pancreatic cancer. Exp. Cell Res..

[100] Qi R., Bai Y., Li K., Liu N., Xu Y., Dal E., Wang Y., Lin R., Wang H., Liu Z. (2023). Cancer-associated fibroblasts suppress ferroptosis and induce gemcitabine resistance in pancreatic cancer cells by secreting exosome-derived ACSL4-targeting miRNAs. Drug Resist. Updat..

[101] Noda K., Sato Y., Okada Y., Nishida K., Kawano Y., Tanahashi T., Bando M., Okamoto K., Takehara M., Sogabe M. (2024). Exosomal miR-199a-3p Secreted from Cancer-Associated Adipocytes Promotes Pancreatic Cancer Progression. Cancer Med..

[102] Chi Y., Xin H., Liu Z. (2021). Exosomal lncRNA UCA1 Derived from Pancreatic Stellate Cells Promotes Gemcitabine Resistance in Pancreatic Cancer via the SOCS3/EZH2 Axis. Front. Oncol..

[103] Fu R., Shao Q., Yang B., Chen Y., Ye Q., Chen X., Zhu J. (2022). MiR-520a-5p/PPP5C regulation pattern is identified as the key to gemcitabine resistance in pancreatic cancer. Front. Oncol..

[104] Zeng Z., Zhao Y., Chen Q., Zhu S., Niu Y., Ye Z., Hu P., Chen D., Xu P., Chen J. (2021). Hypoxic exosomal HIF-1alpha-stabilizing circZNF91 promotes chemoresistance of normoxic pancreatic cancer cells via enhancing glycolysis. Oncogene.

[105] Wang H., Min J., Xu C., Liu Y., Yu Z., Gong A., Xu M. (2023). Hypoxia-elicited Exosomes Promote the Chemoresistance of Pancreatic Cancer Cells by Transferring LncROR via Hippo Signaling. J. Cancer.

[106] Zhou X., Yan Y., Shen Y., Xu M., Xu W. (2024). Exosomes: Emerging Insights into the Progression of Pancreatic Cancer. Int. J. Biol. Sci..

[107] Zhou B., Lei J.H., Wang Q., Qu T.F., Cha L.C., Zhan H.X., Liu S.L., Hu X., Sun C.D., Guo W.D. (2022). Cancer-associated fibroblast-secreted miR-421 promotes pancreatic cancer by regulating the SIRT3/H3K9Ac/HIF-1alpha axis. Kaohsiung J. Med. Sci..

[108] Cao W., Zeng Z., He Z., Lei S. (2021). Hypoxic pancreatic stellate cell-derived exosomal mirnas promote proliferation and invasion of pancreatic cancer through the PTEN/AKT pathway. Aging.

[109] Binenbaum Y., Fridman E., Yaari Z., Milman N., Schroeder A., Ben David G., Shlomi T., Gil Z. (2018). Transfer of miRNA in Macrophage-Derived Exosomes Induces Drug Resistance in Pancreatic Adenocarcinoma. Cancer Res..

[110] Aspe J.R., Diaz Osterman C.J., Jutzy J.M., Deshields S., Whang S., Wall N.R. (2014). Enhancement of Gemcitabine sensitivity in pancreatic adenocarcinoma by novel exosome-mediated delivery of the Survivin-T34A mutant. J. Extracell. Vesicles.

[111] Jia X., Xi J., Tian B., Zhang Y., Wang Z., Wang F., Li Z., Long J., Wang J., Fan G.H. (2024). The Tautomerase Activity of Tumor Exosomal MIF Promotes Pancreatic Cancer Progression by Modulating MDSC Differentiation. Cancer Immunol. Res..

[112] Mikamori M., Yamada D., Eguchi H., Hasegawa S., Kishimoto T., Tomimaru Y., Asaoka T., Noda T., Wada H., Kawamoto K. (2017). MicroRNA-155 Controls Exosome Synthesis and Promotes Gemcitabine Resistance in Pancreatic Ductal Adenocarcinoma. Sci. Rep..

[113] Wang L., Zhang B., Zheng W., Kang M., Chen Q., Qin W., Li C., Zhang Y., Shao Y., Wu Y. (2017). Exosomes derived from pancreatic cancer cells induce insulin resistance in C2C12 myotube cells through the PI3K/Akt/FoxO1 pathway. Sci. Rep..

[114] Yang Z., Zhao N., Cui J., Wu H., Xiong J., Peng T. (2020). Exosomes derived from cancer stem cells of gemcitabine-resistant pancreatic cancer cells enhance drug resistance by delivering miR-210. Cell. Oncol. (Dordr.).

[115] Li Z., Tao Y., Wang X., Jiang P., Li J., Peng M., Zhang X., Chen K., Liu H., Zhen P. (2018). Tumor-Secreted Exosomal miR-222 Promotes Tumor Progression via Regulating P27 Expression and Re-Localization in Pancreatic Cancer. Cell Physiol. Biochem..

[116] Burt A.M., Pallett C.D., Sloane J.P., O’Hare M.J., Schafler K.F., Yardeni P., Eldad A., Clarke J.A., Gusterson B.A. (1989). Survival of cultured allografts in patients with burns assessed with probe specific for Y chromosome. BMJ.

[117] Xu X., Zheng S. (2020). MiR-887-3p Negatively Regulates STARD13 and Promotes Pancreatic Cancer Progression. Cancer Manag. Res..

[118] Schwed-Gross A., Hamiel H., Faber G.P., Angel M., Ben-Yishay R., Benichou J.I.C., Ishay-Ronen D., Shav-Tal Y. (2022). Glucocorticoids enhance chemotherapy-driven stress granule assembly and impair granule dynamics, leading to cell death. J. Cell Sci..

[119] Chen B., Lyssiotis C.A., Shah Y.M. (2025). Mitochondria-organelle crosstalk in establishing compartmentalized metabolic homeostasis. Mol. Cell.

[120] Hu R., Qian B., Li A., Fang Y. (2022). Role of Proteostasis Regulation in the Turnover of Stress Granules. Int. J. Mol. Sci..

[121] Kansanen E., Kivela A.M., Levonen A.L. (2009). Regulation of Nrf2-dependent gene expression by 15-deoxy-Δ^12,14^-prostaglandin J_2_. Free Radic. Biol. Med..

[122] Grabocka E., Bar-Sagi D. (2016). Mutant KRAS Enhances Tumor Cell Fitness by Upregulating Stress Granules. Cell.

[123] Redding A., Fonteneau G., Heinrich S., Gaida M.M., Grabocka E. (2025). Cytosolic Phospholipase A2 Determines Intercellular Heterogeneity of Stress Granules and Chemotherapy Response. Cancer Discov..

[124] Xing F.L., Li B.R., Fang Y.J., Liang C., Liu J., Wang W., Xu J., Yu X.J., Qin Y., Zhang B. (2025). G3BP2 promotes tumor progression and gemcitabine resistance in PDAC via regulating PDIA3-DKC1-hENT in a stress granules-dependent manner. Acta Pharmacol. Sin..

[125] Xing F., Hu Q., Qin Y., Xu J., Zhang B., Yu X., Wang W. (2022). The Relationship of Redox With Hallmarks of Cancer: The Importance of Homeostasis and Context. Front. Oncol..

[126] Kim W.J., Back S.H., Kim V., Ryu I., Jang S.K. (2005). Sequestration of TRAF2 into stress granules interrupts tumor necrosis factor signaling under stress conditions. Mol. Cell Biol..

[127] Zhan T., Betge J., Schulte N., Dreikhausen L., Hirth M., Li M., Weidner P., Leipertz A., Teufel A., Ebert M.P. (2025). Digestive cancers: Mechanisms, therapeutics and management. Signal Transduct. Target. Ther..

[128] Panigrahi D.P., Praharaj P.P., Bhol C.S., Mahapatra K.K., Patra S., Behera B.P., Mishra S.R., Bhutia S.K. (2020). The emerging, multifaceted role of mitophagy in cancer and cancer therapeutics. Semin. Cancer Biol..

[129] Haldipur P., Millen K.J., Aldinger K.A. (2022). Human Cerebellar Development and Transcriptomics: Implications for Neurodevelopmental Disorders. Annu. Rev. Neurosci..

[130] Cui Q., Wang J.Q., Assaraf Y.G., Ren L., Gupta P., Wei L., Ashby C.R., Yang D.H., Chen Z.S. (2018). Modulating ROS to overcome multidrug resistance in cancer. Drug Resist. Updat..

[131] Xing F., Qin Y., Xu J., Wang W., Zhang B. (2023). Stress granules dynamics and promising functions in pancreatic cancer. Biochim. Biophys. Acta Rev. Cancer.

[132] Masoud R., Reyes-Castellanos G., Lac S., Garcia J., Dou S., Shintu L., Abdel Hadi N., Gicquel T., El Kaoutari A., Dieme B. (2020). Targeting Mitochondrial Complex I Overcomes Chemoresistance in High OXPHOS Pancreatic Cancer. Cell Rep. Med..

[133] Azoulay L., Filion K.B., Platt R.W., Dahl M., Dormuth C.R., Clemens K.K., Durand M., Juurlink D.N., Targownik L.E., Turin T.C. (2016). Incretin based drugs and the risk of pancreatic cancer: International multicentre cohort study. BMJ.

[134] Khan H.Y., Kamgar M., Aboukameel A., Bannoura S., Chung B.Y., Li Y., Hallak M.N.A., Philip P.A., Tsai S., Luther S. (2023). Targeting Cellular Metabolism with CPI-613 Sensitizes Pancreatic Cancer Cells to Radiation Therapy. Adv. Radiat. Oncol..

[135] Gampala S., Shah F., Lu X., Moon H.R., Babb O., Umesh Ganesh N., Sandusky G., Hulsey E., Armstrong L., Mosely A.L. (2021). Ref-1 redox activity alters cancer cell metabolism in pancreatic cancer: Exploiting this novel finding as a potential target. J. Exp. Clin. Cancer Res..

[136] Mohan A., Griffith K.A., Wuchu F., Zhen D.B., Kumar-Sinha C., Crysler O., Hsiehchen D., Enzler T., Dippman D., Gunchick V. (2023). Devimistat in Combination with Gemcitabine and Cisplatin in Biliary Tract Cancer: Preclinical Evaluation and Phase Ib Multicenter Clinical Trial (BilT-04). Clin. Cancer Res..

[137] Pardee T.S., Powell B.L., Larson R.A., Maly J., Keng M., Foster M., Choi E.J., Sill H., Cluzeau T., Jeyakumar D. (2024). Devimistat plus chemotherapy vs chemotherapy alone for older relapsed or refractory patients with AML: Results of the ARMADA trial. Blood Neoplasia.

[138] Liu N., Yan M., Tao Q., Wu J., Chen J., Chen X., Peng C. (2023). Inhibition of TCA cycle improves the anti-PD-1 immunotherapy efficacy in melanoma cells via ATF3-mediated PD-L1 expression and glycolysis. J. Immunother. Cancer.

[139] Anderson R., Miller L.D., Isom S., Chou J.W., Pladna K.M., Schramm N.J., Ellis L.R., Howard D.S., Bhave R.R., Manuel M. (2022). Phase II trial of cytarabine and mitoxantrone with devimistat in acute myeloid leukemia. Nat. Commun..

[140] Yang J., Chen F., Fu Z., Yang F., Saba N.F., Teng Y. (2025). Blocking the TCA Cycle in Cancer Cells Potentiates CD36+ T-cell-Mediated Antitumor Immunity by Suppressing ER Stress-Associated THBS2 Signaling. Cancer Res..

[141] Principe D.R., Aissa A.F., Kumar S., Pham T.N.D., Underwood P.W., Nair R., Ke R., Rana B., Trevino J.G., Munshi H.G. (2022). Calcium channel blockers potentiate gemcitabine chemotherapy in pancreatic cancer. Proc. Natl. Acad. Sci. USA.

